# Divergent HIV-1 Restriction Phenotypes of IFITMs Expressed in Target Cells and Incorporated into Virions

**DOI:** 10.3390/biom16030459

**Published:** 2026-03-18

**Authors:** Smita Verma, David Prikryl, Mariana Marin, Ruben M. Markosyan, Andrea Cimarelli, Gregory B. Melikyan

**Affiliations:** 1 Department of Pediatrics, Division of Infectious Diseases, Emory University School of Medicine, Atlanta, GA 30322, USA; smita.verma@emory.edu (S.V.); mmarin@emory.edu (M.M.); 2 Department of Physiology and Biophysics, Rush University, Chicago, IL 60612, USA; ruben_markosyan@rush.edu; 3 Centre International de Recherche en Infectiologie (CIRI), Université de Lyon, Inserm, U1111, Université Claude Bernard Lyon 1, CNRS, UMR5308, École Nationale Supérieure de Lyon, 69007 Lyon, France; andrea.cimarelli@ens-lyon.fr; 4 Children’s Healthcare of Atlanta, Atlanta, GA 30322, USA

**Keywords:** IFITM mutants and chimeras, HIV negative imprinting, target cell protection, viral fusion, subcellular localization

## Abstract

Interferon-induced transmembrane proteins (IFITMs) are broad-spectrum antiviral factors that restrict the entry of many enveloped viruses, including HIV-1, by modifying host membrane properties and trapping fusion at the hemifusion stage. Beyond blocking entry in target cells, IFITMs also reduce the infectivity of virions produced from IFITM-expressing cells, a phenomenon termed “negative imprinting”. Conserved motifs, such as the amphipathic helix and oligomerization motifs, have been reported to be essential for IFITM-mediated protection of target cells from viral infection. Yet, the impact of IFITM incorporation on progeny virion infectivity remains poorly defined. Here, we show that IFITM3 mutants defective in target cell protection activity still markedly impair HIV-1 fusion/infection upon incorporating into virions, without affecting viral maturation or Env incorporation. Immunofluorescence studies suggest mislocalization of the IFITM3 mutants as the reason for the lack of antiviral activity in target cells. Testing the antiviral activity of chimeras between antiviral and non-antiviral IFITM orthologs failed to clearly identify a domain responsible for reduction of HIV-1 infectivity, suggesting that multiple domains may be required for negative imprinting. Interestingly, co-incorporation of non-antiviral dog IFITM1 with human IFITM3 did not interfere with IFITM3’s negative imprinting activity, despite forming mixed hetero-oligomers. This finding implies a dominant, oligomerization-independent antiviral phenotype of IFITM3 in virions. Our findings suggest that IFITMs may protect target cells and negatively imprint progeny virions through distinct mechanisms, underscoring the need to further characterize the molecular basis for the reduced fusion competence of IFITM-containing HIV-1 particles.

## 1. Introduction

Interferon-induced transmembrane (IFITM) proteins are a family of antiviral restriction factors that play a central role in the innate immune response by inhibiting the entry of a broad range of enveloped viruses, including HIV-1 [1,2,3,4,5]. Humans have five IFITMs, of which IFITM1 (hTM1), IFITM2 (hTM2), and IFITM3 (hTM3) exhibit antiviral activity. IFITM proteins are believed to be type II transmembrane proteins, composed of an N-terminal domain (NTD), an “intramembrane” domain (IMD), a conserved intracellular loop (CIL), a transmembrane domain (TMD), and a C-terminal domain (CTD). hTM3 mutagenesis studies have mapped the key conserved domains and residues responsible for the antiviral activity. Motifs essential for blocking the entry of incoming viruses include the amphipathic helix (residues 59–68) within the IMD domain, which modulates lipid packing and spontaneous curvature of cell membranes [6,7], as well as the ^91^GxxxG^95^ motif and residues 73–78 reported to promote hTM3 oligomerization [8,9].

The spectrum of IFITMs’ antiviral activity largely depends on their subcellular localization. The highly homologous hTM2 and hTM3 localize primarily to endo-lysosomal membranes, restricting viruses that utilize endocytic entry pathways, such as Influenza [2], Ebola [10], Zika, and Dengue [3,11] viruses, as well as the laboratory-adapted strains of HIV-1 [12]. In contrast, hTM1 primarily localizes to the plasma membrane and tends to be more effective against viruses that appear to fuse directly at the cell surface, including the Herpes Simplex virus 1 [13], Respiratory Syncytial virus [14], and Measles virus [15,16]. Thus, the relative susceptibility of viruses to restriction by IFITM family members is modulated by the viral entry route [17].

IFITMs exhibit at least two distinct modes of HIV-1 restriction through the viral lifecycle. When expressed in target cells, these proteins inhibit viral fusion by altering the physical properties of host cell membrane that disfavor viral fusion. Specifically, hTM3 expression alters the rigidity and curvature of cell membranes, thereby blocking the transition from hemifusion (characterized by lipid mixing between viral and cellular membranes without content transfer) to a fusion pore [18,19]. The second mode of IFITMs’ antiviral activity discovered by independent laboratories [17,20,21] is through their incorporation into progeny virions, which reduces their infectivity (termed “negative imprinting” [22]). The precise mechanism by which IFITMs reduce the infectivity of HIV-1 and other viruses remains unresolved. While the HIV-1 envelope glycoprotein (Env) is a major determinant of virus’ resistance to target cell protection and negative imprinting by IFITMs, the reasons for such resistance are not well understood. Most HIV-1 Env of primary isolates and transmitted-founder viruses resist hTM3 despite its virion incorporation [23]. The V3 loop of Env is a major determinant of IFITM resistance, likely through regulating Env’s structural stability [23,24]. Early studies reported that hTM3 interacts with HIV-1 Env in producer cells and, thereby, impedes the gp160 precursor cleavage into gp120 and gp41 subunits and Env incorporation into budding HIV-1 particles [21]. However, other studies found no change in Env incorporation into HIV-1 caused by IFITM expression [12,17,25]. Another controversial subject is whether potent negative imprinting requires a robust IFITM incorporation into virions. Although several studies have reported that antiviral potency correlates with the extent of IFITM3 incorporation into virions [1,9,17,25], others found no strict correlation, thus suggesting a complex mechanism of negative imprinting [1,12,26,27].

A fundamental and long-standing question in the field is whether the two modes of antiviral activities of IFITM3 (target cell protection and negative imprinting) are mechanistically linked. Overall, these two restriction modes of mammalian IFITM orthologs appear to correlate, albeit notable exceptions have been reported, including the murine IFITM1 that protects target cells against HIV-1 but lacks significant negative imprinting activity [1,27]. Also, several hTM3 mutations have been reported to preserve the negative imprinting activity, while partially or fully compromising the ability to protect target cells [26]. These findings suggest that these two modes of IFITM-mediated restriction may be uncoupled and driven by distinct mechanisms.

In this study, we asked whether hTM3 residues known to be critical for target cell protection from HIV-1 infection are also essential for negative imprinting of HIV-1. Surprisingly, we found that hTM3 mutants lacking antiviral activity in target cells, including those in the amphipathic helix, the oligomerization motifs, and the C-terminus, significantly impair HIV-1 infectivity when present in the virion. We took advantage of non-antiviral cat IFITM1 (cTM1) and dog IFITM1 (dTM1) orthologs that fail to protect target cells or negatively imprint HIV-1 [27] to construct domain-swapped chimeras with antiviral IFITM orthologs and tested these using cell protection and negative imprinting assays. The overall similar negative imprinting activity of domain-swapped chimeras, which was intermediate between active and inactive orthologs, did not allow us to pinpoint a single determinant responsible for antiviral activity. These results suggest that multiple IFITM domains may contribute to negative imprinting of HIV-1. To probe the role of inter-IFITM interaction/oligomerization in negative imprinting, we co-incorporated hTM3 with the non-antiviral dTM1 into the viral membrane. While these orthologs formed mixed hetero-oligomers in virions, as evidenced by their co-immunoprecipitation, the human IFITM3’s negative imprinting activity remained unchanged. This finding suggests a dominant antiviral phenotype of hTM3 that is independent of homo-oligomerization. Analysis of subcellular localizations of hTM3 mutants lacking cell protection activity indicated that failure to prevent HIV-1 infection correlated with the reduced abundance and/or aberrant localization of the mutants. These results indicate that the lack of cell protection activity was largely driven by mislocalization of the mutant hTM3 proteins, but not by the loss of their capacity to inhibit membrane fusion. Indeed, these mutants retained the ability to interfere with Env-mediated cell-cell fusion. Collectively, our results demonstrate the striking differences in IFITM determinants for the negative imprinting of progeny virions vs. target cell protection, highlighting the need to further dissect the molecular mechanisms that compromise the ability of IFITM-containing HIV-1 to undergo fusion with target cells.

## 2. Materials and Methods

### 2.1. Cell Lines

Human embryonic kidney HEK293T/17 (Cat. #CRL-3216) and human A549 cells (Cat. #CCL-185) were purchased from ATCC (Manassas, VA, USA). HeLa-derived TZM-bl cells (donated by Drs. J.C. Kappes and X. Wu) [28] were received from BEI resources/NIH AIDS Reagent Program (Cat. #HRP-8129, Manassas, VA, USA). HEK293 cells expressing both CD4 and CCR5 were a generous gift from R. Doms (University of Pennsylvania, Philadelphia, PA, USA) [29]. The cells were cultured in high-glucose Dulbecco’s Modified Eagle Medium (DMEM, Cat. #10-013-CV, Corning, NY, USA) supplemented with 10% heat-inactivated fetal bovine serum (FBS, Cat. #SH30071.01HI, HyClone, Logan, UT, USA) and 100 units/mL penicillin/streptomycin (Cat. #SV30010, HyClone, UT, USA), with the recommended selection of antibiotics, as indicated. The HEK293T/17 cells’ growth medium was supplemented with 0.5 mg/mL of Geneticin (Cat. #10131-027, Gibco, ThermoFisher Scientific, Waltham, MA, USA). A549 and HEK293.CD4.CCR5 cells ectopically expressing different IFITM orthologs or hTM3 mutants were obtained by transducing these cells with VSV-G-pseudotyped viruses encoding WT IFITMs, hTM3 mutants, or with the empty pQCXIP vector (Cat. #631514, Clontech Laboratories, Mountain View, CA, USA) and selecting with 1.5 µg/mL puromycin (Cat. #ant-pr-1, InvivoGen, San Diego, CA, USA). Stable cell lines A549.IFITM1, and A549.IFITM3 cells ectopically expressing respective IFITM proteins have been described previously [19].

### 2.2. Reagents, Plasmids, and Antibodies

DMEM without phenol red was obtained from Gibco (Cat. #31053-028, Thermo Fisher Scientific). The Bright-Glo^TM^ luciferase assay system, poly-L-lysine, and poly-D-lysine were purchased and used, as described previously [30]. RIPA buffer (Cat. #ab288006, Abcam, Cambridge, UK), Triton X-100 from Sigma (Cat. #9036-19-5, St. Louis, MO, USA), and Digitonin were purchased from Invitrogen (Cat. #11024-24-1, Research Product International, Mt. Prospect, IL, USA).

The pCAGGS plasmid bearing the full-length HXB2 Env was kindly provided by Dr. J. Binley (Torrey Pines Institute, La Jolla, CA, USA). The GFP-Vpr plasmid was a gift from Dr. T. Hope (Northwestern University, Evanston, IL, USA). The pR9ΔEnvΔNef HIV-1-based packaging vector and pcRev have been described previously [31]. pQCXIP vector-based constructs encoding human IFITM1 (hTM1), IFITM3 (hTM3), and IFITM3-73-78A mutant were a gift from Dr. A.L. Brass (University of Massachusetts Medical School, Worcester, MA, USA) [2]. The pQCXIP-FLAG-IFITM3-Δ59–68, F67Q, G95L, and pQCXIP-IFITM3-G91L were kindly provided by Dr. A. Compton (HIV Dynamics and Replication Program, National Cancer Institute, Frederick, MD, USA) [6,9]. The IFITM3-127-132A mutant, the inactive canine (dog; dTM1), feline (cat; cTM1), oryctolagus (rabbit; R1) IFITM1 proteins, dTM1/D1Lb and cTM1/R1 chimeras cloned into a pcDNA-HA vector were previously described [27]. pNI Cyclophilin A-HiBiT and pMX-pHDomain-HALO-LgBiT (puromycin-selectable retroviral LgBiT vector) were kindly provided by Walther Mothes (Yale University, New Haven, CT, USA) and Z. Matsuda (University of Tokyo, Tokyo, Japan) [32], respectively.

We designed pQCXIP-based constructs HA-cTM1 and HA-dTM1 using forward (5′-GCACCGGTGCCACCATGTACCCATACGATGTTCCAGATTACGCTTATCC-3′) and reverse (5′-CGGAATTCCCAGCGAGCTCTAGCATTTAGGTGACACTATAGAATAGG-3′) primers and cTM1 or dTM1 plasmid as templates, respectively. Fragments were isolated, purified, and, together with the empty pQCXIP vector, digested with AgeI and EcoRI restriction enzymes. Fragments were ligated into a linearized vector, amplified in bacteria, and sequenced after isolation.

The human HIV-1 immunoglobulin (HIV IG) (Cat. #ARP-3957), anti-p24 capture antibody 183-H12-5C (CA183), and Chessie 8 mouse mAb against HIV-1 gp41 (Produced from HIV-1 gp41 Hybridoma cells (Cat. #526)) were obtained from the NIH HIV Reagent Program. Mouse anti-GAPDH antibodies (Cat. #60004-1, Proteintech, Rosemont, IL, USA), rabbit anti-FLAG (Cat. #F7425, Sigma, St. Louis, MO, USA), rabbit anti-HA (Cat. #H6908, Sigma), mouse anti-HA (Cat. #901502, Biolegend, San Diego, CA, USA), rabbit monoclonal [EPR5242] to Fragilis (anti-IFITM3) (Cat. #ab109429, Abcam, Cambridge, UK), rabbit anti-IFITM1 (Cat. #HPA004810, Sigma), mouse anti-GM130 (Cat. #610822, BD Biosciences, San Jose, CA, USA), mouse anti-flag^®^ M2 (Cat. #F1804, Sigma), mouse anti-Human CD63 (Cat. #556019, BD Biosciences), AlexaFluor 568 conjugated Goat anti-Mouse IgG (H + L) (Cat. #A-11004, ThermoFisher), Goat anti-rabbit IgG (H + L) conjugated with Alexa Fluor 647 (Cat. #A-21245, ThermoFisher). The goat anti-HIV gp120, goat anti-human IgG HRP, mouse anti-rabbit IgG HRP, rabbit anti-mouse IgG HRP, donkey anti-goat IgG HRP, and rabbit recombinant antibody for IFITM3 were purchased and used, as previously indicated [30].

### 2.3. Pseudovirus Production, ELISA, and Infectivity Assay

HIV-1 HXB2 Env pseudoviruses were produced by transfecting HEK293T/17 cells using JetPRIME transfection reagent (Cat. #114-15, Polyplus transfection, New York, NY, USA). The cells grown in a 35 mm dish were transfected with 0.6 μg of pCAGGS plasmid expressing HXB2 Env, 0.8 μg of pR9∆Env∆Nef, 0.2 μg of GFP-Vpr, 0.2 μg of pcRev, and 0.5 μg of IFITM constructs or the empty pQCXIP vector. To determine the negative imprinting activity of chimeric IFITMs, the cells were transfected, as above, except that 0.5 μg of different IFITM chimeras replaced the hTM3 constructs. For the virus-cell fusion assays (split NanoLuc luciferase), pseudoviruses were generated similarly, except 0.5 μg pNI Cyclophilin A-HiBiT was substituted for GFP-Vpr in the transfection mix. To produce HIV-1 pseudoviruses for target cell protection assays, HEK293T/17 cells cultured in 35-mm dishes were co-transfected with 0.8 μg of pCAGGS encoding the HXB2 Env, 1.0 μg of NL4-3R–E–Luc, and 0.2 μg of pcRev. For IAV and LASV pseudoviruses (IAVpp and LASVpp), the HIV-1 Env plasmid was substituted with 0.8 μg of pCAGGS expressing H1N1 hemagglutinin (HA) or Lassa virus glycoprotein complex, respectively.

The transfection medium was replaced with phenol-free DMEM/10%FBS after 8–10 h, and the cells were cultured for an additional 36 h, at 37 °C, 5% CO_2._ After this time, the virus-containing culture medium was collected, passed through a 0.45 μm filter, and concentrated 10× using Lenti-X concentrator (Cat. #631232, Takara Bio, San Jose, CA, USA). Following a 3–4 h concentration with Lenti-X, viruses were precipitated by centrifuging at 1500 rcf for 45 min at 4 °C, resuspended in PBS++ (Ca^2+^/Mg^2+^; Cat. #21-030-CM, Corning MediaTech, Manassas, VA, USA), and stored in aliquots at −80 °C.

The p24 content of pseudoviruses was determined by an ELISA assay, as previously described [33]. The virus infectivity was measured by a luciferase reporter expression in TZM-bl cells using the previously published protocol [30]. Luciferase activity was determined by adding Bright-Glo Luciferase substrate and reading the plates with a TopCount NXT luminescence counter (PerkinElmer, Waltham, MA, USA).

### 2.4. Virus–Cell Fusion Assay Using Split NanoLuc Luciferase (NanoLuc) System

Virus–cell fusion was quantified using a split NanoLuc (NanoBiT) complementation assay [34,35]. NanoLuc is a 19-kDa luciferase that has been split into a large fragment (LgBiT, ~18 kDa) and a small high-affinity peptide (HiBiT, ~1.3 kDa).

LgBiT-expressing HEK293.CD4.CCR5 target cells were generated by transduction with VSV-G/MLV-Gag-Pol/pMXs-puro-PH-Halo-LgBiT pseudoviruses and selection with puromycin. For the fusion assay, HEK293.CD4.CCR5.LgBiT cells were seeded in poly-D-lysine–coated, black, clear-bottom 96-well plates (Cat. #4929, ThermoFisher Scientific) in DMEM supplemented with 10% FBS and antibiotics. The following day, confluent monolayers (~1 × 10^5^ cells/well) were pretreated with 10 µM DrkBiT peptide (VSGWALFKKIS, Cat. #SC1208, GenScript, Piscataway, NJ, USA) in DMEM containing 20 mM HEPES for 30–60 min at 37 °C to inhibit extracellular LgBiT activity. Cyclophilin A-HiBiT-tagged pseudoviruses (0.3 pg/µL of p24) were diluted in assay medium containing 10 µM DrkBiT and 1× Endurazine substrate (Cat. #N2570, Promega, Madison, WI, USA) and added to target cells (50 µL/well). Plates were centrifuged at 1550× *g* for 30 min at 12 °C, followed by incubation at 37 °C, 5% CO_2_ for 90 min. Luminescence was measured using a TopCount NXT luminescence plate reader, and background signals from uninfected LgBiT cells were subtracted.

### 2.5. Western Blot and Immunoprecipitation

Equal amounts of pseudoviruses (based on p24 concentration) were boiled using 2× Laemmli buffer (Bio-Rad, Hercules, CA, USA). The samples were loaded onto a 4–15% polyacrylamide gel (Cat. #4561083, Bio-Rad, CA, USA) and transferred onto a 0.45 μm nitrocellulose membrane (Cat. #10600002, Amersham, Cytiva, Marlborough, MA, USA). The membranes were blocked in 10% dry milk in 0.1% Tween 20 in phosphate buffered saline solution and incubated overnight at 4 °C with different primary antibodies as follows: HIV IG (1:1000 dilution), rabbit anti-IFITM1 (1:500 dilution), recombinant anti-fragilis antibody for IFITM3 (1:1000), mouse anti-HIV gp41 (1:100), goat anti-HIV gp120 (1:500), rabbit anti-HA (1:500), and mouse anti-GAPDH (1:2000). Secondary antibodies were either goat anti-human HRP (1:1000), mouse anti-rabbit HRP, rabbit anti-mouse HRP, or donkey anti-goat HRP (1:1000). Incubation with these antibodies was carried out for 1 h at room temperature at a dilution of 1:3000, unless otherwise specified.

For whole cell lysate analyses, producer or target cells were harvested and processed, as described previously [31]. The respective proteins were detected with rabbit anti-IFITM3, rabbit anti-IFITM1, rabbit anti-FLAG, and mouse anti-GAPDH antibodies using a chemiluminescence reagent (Cat. #RPN2232, Cytiva, Marlborough, MA, USA).

For co-immunoprecipitation, the extracts from HEK293T/17 producer cells were prepared using RIPA buffer without SDS, supplemented with the complete protease inhibitor cocktail (Cat. #11836153001, Roche Applied Science, Indianapolis, IN, USA). The lysates were then centrifuged at 1500× *g* for 5 min to pellet the nuclei. The total protein concentrations in the extracts were determined using a micro-BCA protein assay kit (Cat. #PI23235, ThermoFisher Scientific, MA, USA). The lysates were pre-cleared by incubating with pre-washed protein A/G agarose suspension (Cat. #IP05, Millipore Sigma, Burlington, MA, USA) for 1 h at 4 °C with gentle rotation, followed by centrifugation at 1500× *g* for 5 min. The supernatant was transferred to a new tube. The rabbit anti-HA antibody (typically 1.5 µg per 200 µL of lysate) was added to the lysate and incubated overnight at 4 °C. A suspension of pre-washed protein A/G beads was then added to the lysate, and the mixture was incubated for 1 h at room temperature with gentle rotation. The beads were washed twice with RIPA buffer without SDS, by centrifuging at 3000–4000× *g* for 5 min to pellet the beads. Proteins were eluted and denatured by resuspending the beads in 2× Laemmli buffer and heating at 95 °C for 5 min. The eluted proteins were subjected to SDS-PAGE using 4–15% gels. Reference samples corresponding to 15–17 µg of total cell lysates were also loaded onto the gel. Proteins were transferred electrophoretically to a nitrocellulose membrane, which was subsequently blocked and probed using rabbit anti-HA (1:500), rabbit anti-IFITM3 (1:1000), and mouse anti-GAPDH (1:2000) antibodies. Mouse anti-rabbit HRP and rabbit anti-mouse HRP were used as secondary antibodies and incubated for 1 h at room temperature at a 1:3000 dilution.

The chemiluminescence signal was recorded on ChemiDoc XRS+ (Bio-Rad) using Image Lab software version 5.1. Densitometry analysis was performed using Image Lab Software, version 6.1 (Bio-Rad Laboratories, Inc.). Unless stated otherwise, densitometric analysis was done on triplicate samples.

### 2.6. Cell-Cell Fusion Assay

HEK293T/17 effector cells and TZM-bl target cells were maintained under standard culture conditions. Effector cells were transfected with HXB2 Env (1.5 μg) and pcRev (0.5 μg) expressing plasmids, and target cells were transfected with WT or mutant IFITM constructs (2 μg) using jetPRIME transfection reagent. Forty-eight hours post-transfection, effector cells were loaded with Calcein AM (Cat. #C3099, ThermoFisher) and target cells with CMAC Blue (Cat. #C2110, ThermoFisher). Cells were then detached with PBS containing 0.5 mM EDTA/EGTA, mixed at a 1:1 ratio, and seeded onto poly-L-lysine–coated chamber slides (Cat. #177402, Lab-Tek, Naperville, IL, USA). After incubation at 37 °C for 2 h, fusion activity was assessed by monitoring dual-labeled cell fusion products using a fluorescence microscope (Axiovert 100A; Carl Zeiss, Thornwood, NY, USA), as described previously [36].

### 2.7. Flow Cytometry

HeLa TZM-bl cells (~1 × 10^6^) expressing hTM3 or mutant constructs were immunostained for surface CD4 or CXCR4. Cells were incubated with human anti-CD4 (clone SIM2, Cat. #723) or mouse anti-CXCR4 (clone 12G5, Cat. #3439) monoclonal antibodies (NIH HIV Reagent Program), each diluted 1:100 in PBS++ supplemented with 5% goat serum and 0.1% sodium azide, for 45 min on ice. After washing with the same buffer, cells were incubated with the corresponding 1:500 diluted secondary antibodies-FITC-conjugated goat anti-human IgG (Cat#F9887, Sigma) or Alexa Fluor 488-conjugated goat anti-mouse IgG (Cat. #A10667, ThermoFisher) for 45 min on ice. The cells were washed, resuspended in 800 µL PBS−−, and analyzed on a Guava EasyCyte flow cytometer (Luminex, Hayward, CA, USA).

### 2.8. Immunostaining, Microscopy, and Image Analysis

Cells were plated in 8-well chamber coverslips (Cat. #155411, Nunc, Rochester, NY, USA) coated with 0.2 mg/mL collagen (Cat. #C9791, Sigma) one day before imaging. The next day, cells were fixed with 4% PFA (Cat. #28906, ThermoFisher Scientific) for 20 min at room temperature, permeabilized with 150 µg/mL digitonin or 0.1% Triton X-100 for 20 min, and blocked with 10% FBS for 30 min. Cells were next incubated with respective primary antibodies diluted in 10% FBS for 1.5 h, washed, and incubated with secondary antibodies in 10% FBS for 45 min. Samples were stained with Hoechst 33342 (4 µM, Cat. #H3570, Invitrogen) in PBS for 5–10 min before imaging.

Images were acquired on a Zeiss LSM 880 confocal microscope using a plan-apochromat 63×/1.4NA oil objective. The entire cell volume was imaged by collecting multiple Z-stacks. For colocalization analysis, maximum intensity projections were generated from five medial z-slices, selected consistently across all samples. IFITM3 signal overlap with GM130 was quantified using Mander’s and Pearson’s overlap coefficients calculated with the JaCoP FIJI plugin [37].

### 2.9. Statistical Analysis

Unless noted otherwise, statistical tests were performed using unpaired Student’s *t*-test implemented in GraphPad Prism version 9.3.1 for Windows (GraphPad Software8.0.1, La Jolla, CA, USA). ns (*p* > 0.05); * (*p* < 0.05); ** (*p* < 0.01); *** (*p* < 0.001).

## 3. Results

### 3.1. hTM3 Mutants Lacking Target Cell Protection Activity Negatively Imprint HIV-1

**Figure 1:**
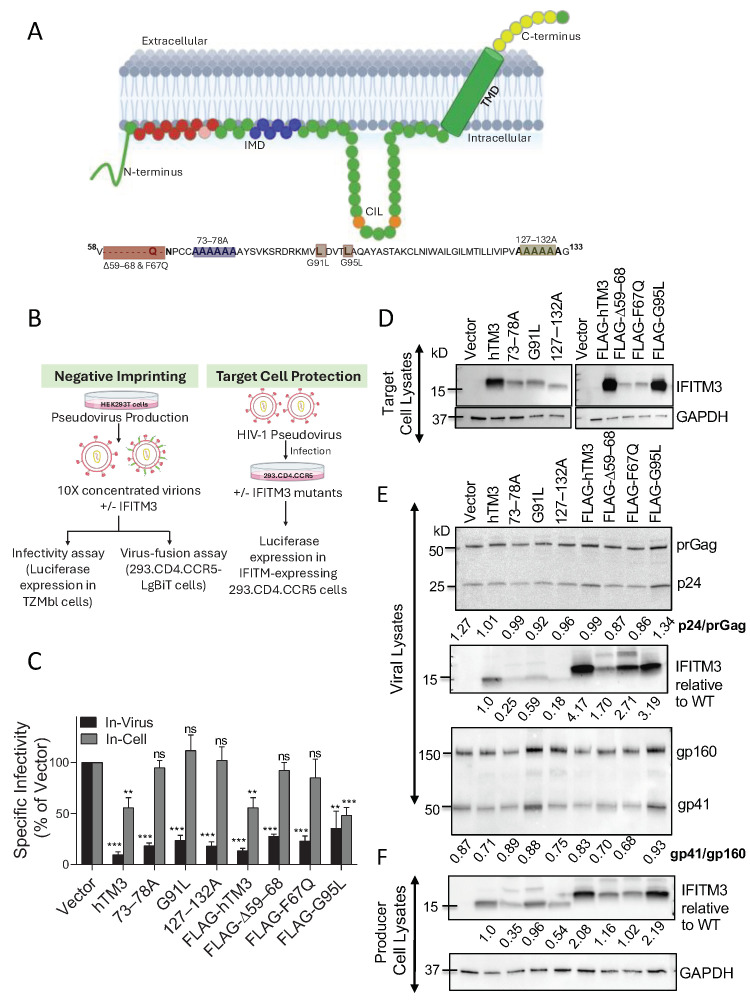
hTM3 mutants drive negative imprinting of HIV-1 virions but fail to confer target-cell resistance. (**A**) Schematic representation of the human IFITM3 (hTM3) protein highlighting the engineered mutations. The amino acid sequence spanning residues 58–133 is shown below, with highlighted mutations in different colors. Specific substitutions used in this study are marked with distinct colors to illustrate different hTM3 mutants. (**B**) Overview of the experimental approaches used to elucidate the anti-HIV-1 activity of IFITMs against HIV-1. Target-cell protection was assessed using infectivity assays in HEK293.CD4.CCR5 cells stably expressing hTM3/mutant (Left). Effects of IFITM expression in HEK293T/17 virus-producing cells on HIV-1 infectivity were measured in TZM-bl reporter cells (Right). (**C**) The impact of hTM3 and mutant proteins on HIV-1 infectivity was evaluated both “in-virus” (measuring negative imprinting of HIV-1 by intraviral IFITMs using target TZM-bl cells) and “in-cell” (measuring protection of HEK293.CD4.CCR5 cells against HIV-1 infection). Target cells were infected for 48 h with indicated pseudoviruses. The infectivity was assessed by measuring the luciferase activity and normalized to their p24 content. Statistical significance was determined using Student’s *t*-test. ns (*p* > 0.05); ** (*p* < 0.01); *** (*p* < 0.001). (**D**–**F**) Western blot analysis of hTM3 and viral protein expression in target cells and in virions. (**D**) IFITM expression in transduced HEK293.CD4.CCR5 target cells (**E**) Viral lysates were probed for Gag precursor prGag and p24, Env incorporation and cleavage, and for IFITM3 incorporation into virions. (**F**) Expression levels of hTM3 and its mutant constructs were also analyzed in producer HEK293T/17 cells. Original Western Blot images are provided in Supplementary Figures S14–S16.

We first sought to compare the ability of hTM3 and its mutants to protect target cells from HIV-1 infection and to negatively imprint progeny virions. We selected several loss-of-function hTM3 mutants spanning different domains, using a combination of untagged and FLAG-tagged constructs (Figure 1A). Since N-terminal FLAG-tagging increases the expression levels of unstable hTM3 mutants, such as Δ59–68, F67Q, and G95L (Supplementary Figure S1A), we intentionally used the FLAG-tagged versions of these mutants to ensure a meaningful comparison across variants. The Δ59–68 and F67Q mutations within the IMD domain delete or disrupt the conserved amphipathic helix, respectively, as well as destroy the cholesterol binding site [6,38]. The 73–78A (a 6-alanine substitution) and G91L and G95L mutations have been reported to impair the hTM3 homo-oligomerization [3,8,9], which appears more critical for target cell protection than for restricting virion infectivity [9]. The 6-alanine substitution of the C-terminal 127–132 residues (denoted 127–132A) diminishes target cell protection, yet retains negative-imprinting activity, indicating that this C-terminal region may differentially regulate these two antiviral functions [26].

Target cell protection was assessed using HEK293 cells stably expressing CD4 and CCR5, along with hTM3 or its mutants (endogenous expression of CXCR4 makes these cells conducive to infection by CXCR4-tropic HIV-1). Cells were infected with an NL4-3–based HIV-1 reporter virus pseudotyped with HXB2 Env. In parallel, negative imprinting of virions was examined by producing independent panels of HIV-1 pseudoviruses in HEK293T/17 cells expressing wild-type or mutant hTM3. Each panel included three untagged and three N-terminally FLAG-tagged mutants, along with the corresponding untagged/tagged hTM3 controls (Figure 1B).

Expression of hTM3 in target cells reduced HIV-1 infection by approximately two-fold (Figure 1C, gray bar). In agreement with the previously published studies [6,26], the hTM3 mutants did not display significant antiviral activity in target cells, except for the FLAG-tagged G95L mutant, which was expressed at a high level (see below) and significantly reduced single-cycle HIV-1 infection. Western blot analysis of target cell lysates showed variable expression levels among hTM3 mutants, which were less abundantly expressed than the respective hTM3 control or the FLAG-G95L mutant (Figure 1D). Control experiments measuring the effect of hTM3 variants expressed in target cells on infectivity of pseudoviruses decorated with the Influenza A virus (IAV) hemagglutinin (HA) revealed a strong inhibition of IAV HA pseudovirus entry by WT, whereas the mutants exhibited no or little antiviral activity (Supplementary Figure S2A), in agreement with the previous reports [6,9,26,38]. As expected, neither hTM3 nor the hTM3 mutants inhibited virus entry mediated by the IFITM3-resistant Lassa virus (LASV) glycoprotein (Supplementary Figure S2B) [2,4].

Next, we evaluated the ability of wild-type and mutant hTM3 proteins to modulate the infectivity of HIV-1 pseudoviruses produced by transfection of HEK293T cells using TZM-bl cells as targets (Figure 1C, black bars). Consistent with previous studies [17,25,30,39], the infectivity of HXB2 Env pseudoviruses containing hTM3 was reduced by 8.64 ± 0.5 fold. Remarkably, mutants that failed to protect target cells from HIV-1 infection significantly (3–8-fold) diminished the infectivity upon incorporation into virions. We also assessed the impact of hTM3 and selected mutants on pseudoviruses bearing the macrophage-tropic AD8 Env that is resistant to hTM3 restriction [24]. As anticipated, AD8 Env was not sensitive to restriction by hTM3 or its mutants (Supplementary Figure S3A), despite their robust incorporation into pseudovirions (Supplementary Figure S3B). These results confirm the previous reports [23,24,30] that HIV-1 Env is a major determinant of hTM3 resistance.

**Figure 2:**
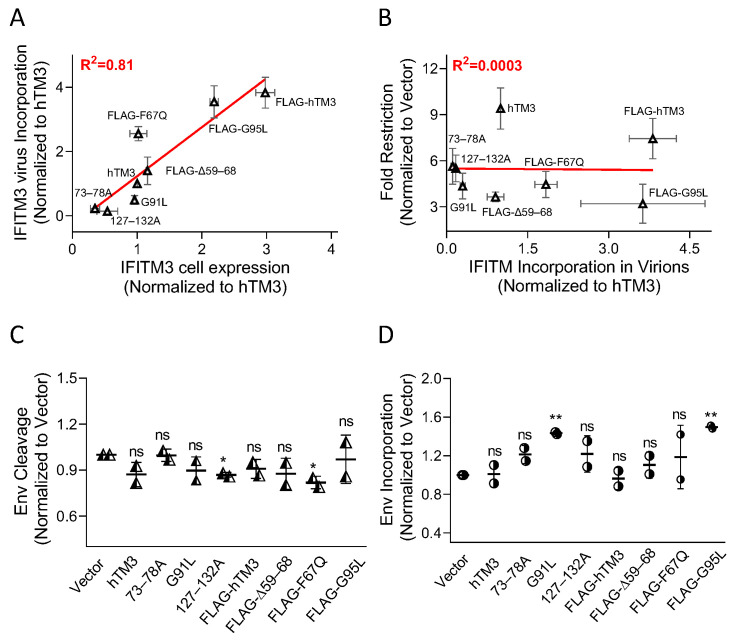
Negative imprinting by hTM3 mutants does not correlate with hTM3 incorporation or Env cleavage or incorporation. (**A**) Correlation between WT and mutant hTM3 expression in virus-producing cells and incorporation into virions. (**B**) Correlation between incorporation into virions and antiviral activity for untagged and N-terminally FLAG-tagged hTM3 constructs. Linear regression was performed using GraphPad Prism. The best-fit regression line (red) and R^2^ value are indicated. (**C**,**D**) Analysis of the extent of Env cleavage (gp41/gp160 ratio, (**C**)) and Env incorporation (the ratio of gp41 + gp160 bands over p24 + prGag bands, (**D**)) in immunoblots of viral lysate. The data in panels (**A**–**D**) are averages from two independent viral preparations. Statistical significance was determined using Student’s *t*-test. ns (*p* > 0.05); * (*p* < 0.05); ** (*p* < 0.01).

Immunoblot analyses of viral (Figure 1E) and producer cell lysates (Figure 1F) revealed distinct levels of expression in cells and virus incorporation among the hTM3 mutants. hTM3/FLAG-hTM3 were better expressed in cells and incorporated into virions than the mutants, except for FLAG-G95L. The incorporation of hTM3 mutants into HIV-1 correlated with their expression levels in virus-producing cells (R^2^ = 0.82; Figure 2A). Accordingly, FLAG-tagged hTM3 variants showed enhanced expression and virion incorporation compared with their untagged counterparts (Figure 1E,F and Figure S1B,C). Immunoblotting revealed that incorporation of different hTM3 mutants did not noticeably affect the virus production or Gag cleavage across independent viral preparations (Figure 1E and Figure S4).

We next sought to test the link between hTM3 incorporation into virions determined by Western blotting and the antiviral phenotype expressed as fold-restriction (Figure 2B). Consistent with previous reports [4,26], we found no correlation between fold-restriction and incorporation levels of wild-type and the mutants hTM3, indicating that the amount of hTM3 in HIV-1 particles is not the primary determinant of negative imprinting. Next, we assessed if negative imprinting by a given hTM3 variant exhibits dose-dependence by titrating the amounts of hTM3 or FLAG-Δ59–68 (randomly picked from the panel of mutants) plasmids used to transfect the virus-producing HEK293T cells. Increasing the quantity of either plasmid elevated the cellular levels of hTM3 and the FLAG-Δ59–68 mutant and improved their incorporation into virions (Supplementary Figure S5B,D). Importantly, we observed a concomitant increase in negative imprinting for both hTM3 and FLAG-Δ59–68 mutant (Supplementary Figure S5A,C). Immunoblot analysis did not reveal considerable changes in Env processing and incorporation into virions caused by the increased expression of the hTM3 and its mutant (Supplementary Figure S5B,D). Thus, although no correlation between negative imprinting and the levels of virus incorporation was observed for different hTM3 variants, the negative imprinting activity of individual IFITMs (hTM3 and FLAG-Δ59–68) appears to scale with their intraviral levels.

To test whether the reduced infectivity of IFITM-containing HXB2 pseudoviruses resulted from impaired Env processing or incorporation into virions, we performed densitometric analysis of immunoblots. Analysis of two independent viral preparations revealed variable Env cleavage (gp41/gp160 ratio) among virions carrying hTM3 and its mutants (Figure 2C). Analysis of Env incorporation (gp41 + gp160/p24 + prGag) into virions for hTM3 and its mutants showed no significant reduction (Figure 2D; Note an increase in Env signal for the G91L and FLAG-G95L mutants). While most mutants had a minimal impact on Env cleavage, 127–132A and F67Q incorporation significantly reduced Env processing, suggesting that this effect may contribute to HIV-1 restriction by these mutants.

### 3.2. hTM3 Mutants Expressed in Target or Effector Cells Inhibit HIV-1 Env-Mediated Membrane Fusion

**Figure 3:**
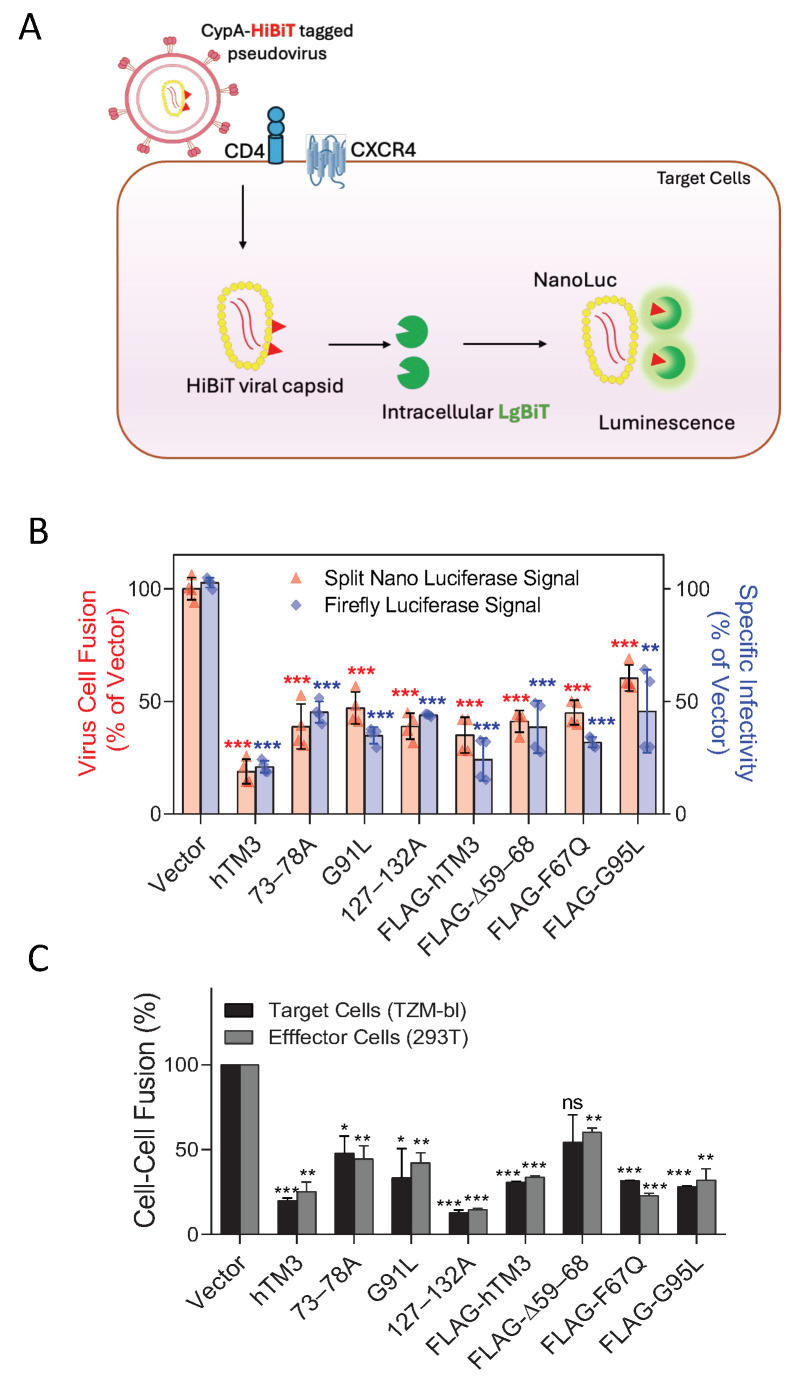
hTM3 mutants that fail to protect target cells against HIV-1 infection inhibit pseudovirus-cell fusion and Env-mediated cell-cell fusion. (**A**) Schematic representation of the split Nano-Luciferase (Nano-Luc) system. The small HiBiT peptide binds to the complementary LgBiT fragment, reconstituting an active NanoLuc enzyme. NanoLuc components are depicted out of scale for illustrative clarity. (**B**) Assessment of the impact of hTM3 mutants on HIV-1 infectivity (negative imprinting) and the ability of HXB2 pseudovirus to fuse to target cells. Viral infectivity was measured in reporter TZM-bl cells based upon luciferase expression (blue), while virus–cell fusion was directly measured using a split NanoLuc assay (red). Despite the lack of target-cell protection by the selected hTM3 mutants, these proteins potently inhibited virus-cell fusion, as revealed by a significant reduction in NanoLuc signal. (**C**) Cell-cell fusion assay. HEK293T/17 cells transiently expressing HIV-1 Env (HXB2 strain) were fused to TZM-bl cells expressing CD4 and coreceptors. hTM3 and its mutants were expressed by transfection of either effector HEK293T/17 cells or target TZM-bl cells. Effector and target cells loaded with calcein AM and CMAC cytoplasmic dyes, respectively, were co-cultured for 2 h at 37 °C, and the extent of fusion (fraction of double-positive cells) was measured by fluorescence microscopy. Statistical significance was determined using Student’s *t*-test. ns (*p* > 0.05); * (*p* < 0.05); ** (*p* < 0.01); *** (*p* < 0.001).

To directly assess the impact of IFITM incorporation into HIV-1 particles, we measured the virus’s ability to undergo fusion with target cells, using a split NanoLuc assay [34,35]. The split NanoLuc consists of two complementary domains—a small HiBiT fragment (~1.3 kDa) engineered for high-affinity binding to the large LgBiT fragment (~18 kDa) [34,40]. We created HEK293 target cells stably expressing CD4, CCR5, and LgBiT. Infection with CypA–HiBiT–tagged pseudoviruses enabled sensitive quantification of virus–cell fusion based upon the resulting luminescence signal of the reconstituted NanoLuc enzyme (Figure 3A). We detected a consistent reduction in the fusion competence of pseudoviruses containing hTM3 or its mutants (Figure 3B). The nearly identical fold-restriction of viral fusion and infection (compare Figure 1C and Figure 3B) further supports the notion that virion-incorporated hTM3 and its mutants inhibit the fusion step of HIV-1 entry. Collectively, our findings reveal distinct anti-HIV phenotypes of hTM3 mutants, depending on whether they are expressed in target cells or incorporated into virions.

To investigate whether the distinct phenotypes of hTM3 in target cells and virions are due to the differences in the properties of cellular and viral membranes, we employed a cell-cell fusion assay. It has been previously shown that all three human IFITM proteins (hTM1, hTM2, and hTM3), when overexpressed in cells, potently inhibit cell-cell fusion mediated by diverse viral glycoproteins [18,21]. Thus, regardless of distinct subcellular localizations of hTM1 vs. hTM2/hTM3 (plasma membrane vs. late endosomes, respectively), overexpressed IFITMs appear to be present on the cell surface and interfere with viral protein-mediated cell fusion. Strikingly, hTM3 mutants that were inactive in target cell protection potently inhibited HIV-1 Env–mediated cell-cell fusion, irrespective of whether they were expressed in target or Env-expressing effector cells (Figure 3C). Of note, the FLAG-Δ59–68 mutant was the least potent in inhibiting cell-cell fusion (~50%), and its impact on fusion upon expression in target cells did not reach significance. As shown in Supplementary Figure S6, and in agreement with previously published results [26], inhibition of Env-mediated cell-cell fusion was not due to a reduction in CD4 or CXCR4 expression by hTM3 or its mutants expressed in target cells. This differential impact of mutations on target cell protection vs. negative imprinting of virions and cell-cell fusion implies that the mutant hTM3s have not lost the ability to inhibit Env-mediated membrane fusion and that the viral membrane is not necessarily more conducive to IFITM-mediated restriction of fusion than the target cell membrane.

### 3.3. hTM3 Mutants Exhibit Altered Subcellular Localizations

**Figure 4:**
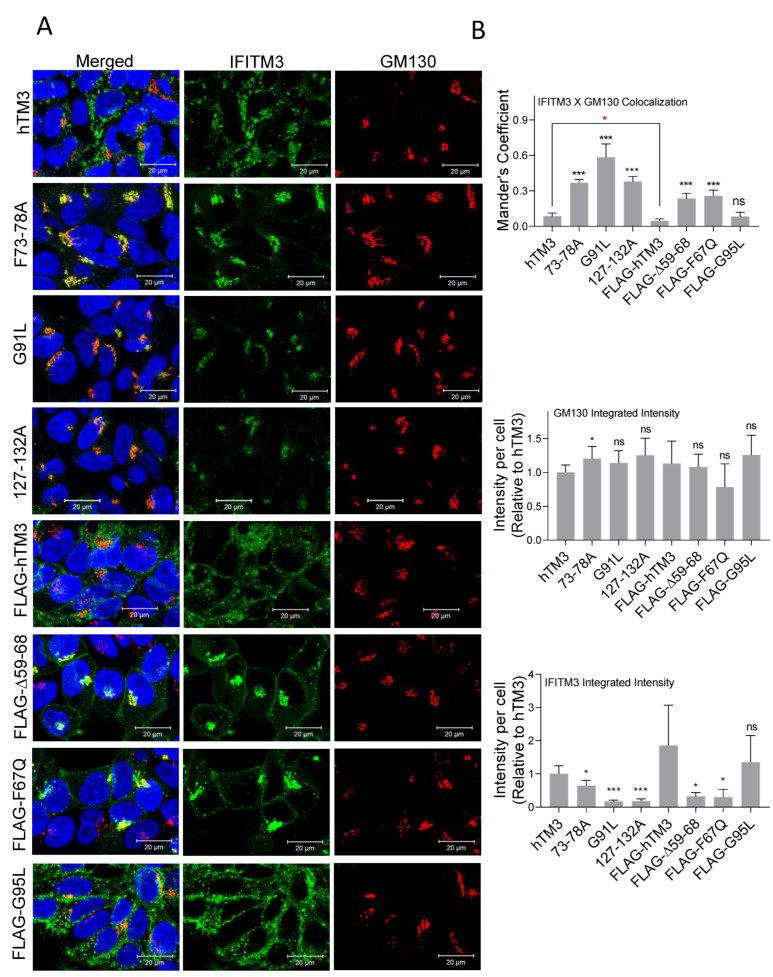
Abundance and subcellular localization of hTM3 mutants. (**A**) HEK293.CD4.CCR5 cells stably expressing the indicated hTM3 mutants were fixed, permeabilized with Digitonin, and immunostained with anti-IFITM3 (green) and anti-GM130 (Golgi, red) antibodies. Nuclei were counterstained with Hoechst-33342 (blue). Images shown represent maximum intensity projections of five medial z-slices. Scale bar: 20 μm. (**B**) Quantitative analysis of IFITM3 expression levels was performed using integrated fluorescence intensities per cell, normalized to hTM3 signal. Colocalization with GM130 was assessed on maximum intensity projections using Mander’s overlap coefficient (see Supplementary Figure S7 for colocalization analysis using Pearson’s overlap coefficient). Statistical analyses were performed for untagged mutants relative to untagged hTM3 and for FLAG-tagged mutants relative to FLAG-hTM3 (black asterisks) and between untagged and tagged hTM3 (red asterisks). Data represent mean ± SD from multiple fields of view across two independent experiments. Statistical significance was determined using Student’s *t*-test. ns (*p* > 0.05); * (*p* < 0.05); *** (*p* < 0.001).

We examined a possible link between hTM3 localization and cell protection from HIV-1 infection by imaging untagged and N-terminally FLAG-tagged hTM3 mutants in digitonin-permeabilized and fixed HEK293.CD4.CCR5 target cells. The hTM3 mutants, 73–78A, G91L, 127–132A, FLAG-Δ59–68, and FLAG-F67Q, were less abundantly expressed and appeared to exhibit altered cellular localizations compared to hTM3, as evidenced by their higher colocalization with the cis-Golgi marker GM130 (Figure 4A,B and Figure S7) [40,41]. This finding is consistent with previous reports describing aberrant Golgi retention of hTM3 mutants, including the 73–78A mutant (Figure 4A,B) [8,42]. The FLAG-G95L mutant was an exception, as its expression and distribution were comparable to tagged and untagged hTM3 (Figure 4A,B and Figure S7), which correlated with its nearly 2-fold restriction of HIV-1 infection of these cells (Figure 1C).

To directly assess the impact of N-terminal FLAG-tagging, we compared subcellular localization and the cell protection activity of tagged and untagged versions of hTM3 and the ∆59–68 and F67Q mutants expressed in target cells. In agreement with the immunoblot data (Supplementary Figure S1), we observed that N-terminal FLAG tagging increased the expression levels of the hTM3 mutants (Supplementary Figure S8A). Importantly, FLAG tagging did not alter the subcellular localization patterns of the mutants (Supplementary Figure S8B), allowing for a direct comparison of their antiviral activities. While expression of both FLAG-tagged and untagged hTM3 reduced HIV-1 infection by approximately two-fold, the selected tagged or untagged hTM3 mutants did not significantly protect target cells from HIV-1 infection (Supplementary Figure S8C).

We have previously reported that digitonin permeabilization allowed antibody access to a subset of IFITM proteins, whereas a harsher cell permeabilization by TX-100 reveals a late endosome-resident pool of these molecules colocalized with the late endosome marker CD63 [43]. However, immunofluorescence staining of HEK293.CD4.CCR5 cells permeabilized with TX-100 for hTM3 and CD63 did not produce conclusive results due to the strong modulation of CD63 expression levels upon expression of hTM3 variants (Supplementary Figure S9A,B). Note that the elevation of CD63 levels in hTM3 overexpressing cells has been previously reported [26]. Collectively, these results support the altered subcellular distribution of the hTM3 mutants in target cells and suggest that their mislocalization may be linked to the apparent loss of antiviral activity. At the same time, the ability to negatively imprint virions and block cell–cell fusion implies that these hTM3 mutants retain the ability to interfere with HIV-1 mediated fusion, when localized in the proximity to Env or cognate HIV-1 receptors.

### 3.4. Multiple IFITM Domains Are Likely Required for Negative Imprinting of HIV-1

Given the lack of correlation between the ability of IFITM mutants to protect cells from HIV-1 infection and negative imprinting of progeny virions (Figure 1C), we sought to delineate the functional domains responsible for negative imprinting. We have previously characterized the ability of mammalian IFITM1 orthologs, including rabbit (R1), cat (cTM1), and dog (D1Lb & dTM1), and other IFITM proteins to negatively imprint HIV-1 [27]. Among these, R1 and D1Lb exhibited robust antiviral activity, whereas cTM1 and dTM1 lacked antiviral activity in both target cell protection and negative imprinting assays. Using N-terminally HA-tagged constructs, we reaffirmed the inactivity of cTM1 and dTM1 and examined a panel of domain-swamped IFITM1 chimeras between active and inactive orthologs.

Whereas hTM1 incorporation into progeny virions caused a 3-fold reduction in infectivity relative to the vector control, cTM1 and dTM1 failed to reduce virion infectivity (Supplementary Figure S10A), as expected [27]. Western blot analysis verified efficient expression and incorporation of hTM1 into virions, even when using a relatively low amount of plasmid for transfection (0.2 µg), whereas reduced levels of cTM1 and dTM1 were detected in both virions (Supplementary Figure S10B) and target cells (Supplementary Figure S10C), in agreement with our earlier findings [27]. These differences in incorporation were not attributable to altered viral protein synthesis or virus production, judging by the largely unchanged expression/cleavage of Gag and Env across the IFITM1 orthologs (Supplementary Figure S10B).

**Figure 5:**
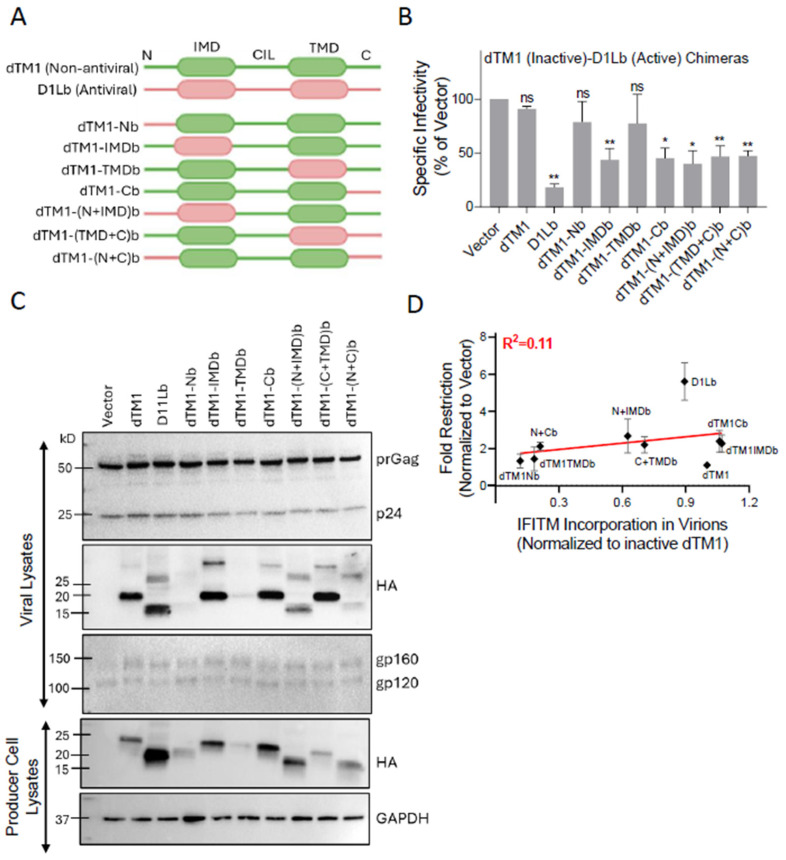
Design, expression, and negative imprinting activities of chimeras between active and inactive dog IFITM1 (dTM1) orthologs. (**A**) Schematic representation of the chimeras with swapped domains of dTM1 (non-antiviral) and D1Lb (antiviral) constructs. (**B**) Normalized infectivity of HIV-1 pseudoviruses carrying the indicated IFITM1 chimeras in TZM-bl target cells. Cells were infected for 48 h with pseudovirions produced by HEK293T/17 cells transfected with the respective chimeric constructs. Infectivity was measured using a luciferase assay and normalized to the vector control. Data represent the mean ± SD for three independent viral preparations. Statistical significance was determined using Student’s *t*-test. ns (*p* > 0.05); * (*p* < 0.05); ** (*p* < 0.01). (**C**) Western blot analyses showing Gag processing (p24/prGag), HA-IFITM incorporation, and HIV-1 Env cleavage (gp120/gp160 ratio) and incorporation into virions, along with HA-IFITM expression levels of the chimeric constructs in HEK293T/17 producer cell lysates (bottom). Original Western Blot images are provided in Supplementary Figure S17. (**D**) Correlation between fold-restriction and IFITM incorporation into virions.

Having validated the active and inactive IFITM orthologs, we analyzed a panel of domain-swapped N-terminally HA-tagged IFITM1 chimeras between antiviral (R1, D1Lb) and non-antiviral (cTM1, dTM1) homologs (Figure 5A and Figure S11A). The cell protection activities of these chimeras have been previously characterized, but their ability to negatively imprint HIV-1 was not systematically investigated [27]. Consistent with our published data [27], incorporation of D1Lb caused a robust (5.5-fold) reduction in virion infectivity. Nearly all chimeras, except dTM1-Nb and dTM1-TMDb, which did not exhibit antiviral activity, were moderately restrictive and less potent than D1Lb (Figure 5B). Immunoblotting for p24 confirmed comparable Gag processing across samples (Figure 5C), and HA-IFITM blots indicated efficient incorporation of most chimeras, except for dTM1-Nb, dTM1-TMDb, and dTM1-(N+C)b, which showed weaker bands in both viral and producer cell lysates (Figure 5C). Env incorporation and cleavage were largely unaffected across the panel (Figure 5C). The lack of negative imprinting by dTM1-Nb and dTM1-TMDb tracked with their nearly undetectable incorporation into virions (Figure 5C). However, fold-restriction across all chimeras did not correlate with IFITM incorporation into virions, reaffirming that the antiviral potency is somewhat independent of the level of IFITMs in virions (Figure 5D). The similar fold-restriction across the panel of well-expressed chimeras indicates that no single IFITM1 domain is critical for negative imprinting.

The cTM1/R1 chimeras showed similar overall phenotypes to dTM1/D1Lb chimeras (Supplementary Figure S11A). Incorporation of the active R1 ortholog reduced virion infectivity by 2.4-fold, while cTM1 was without an effect (Supplementary Figure S11B). Most chimeras exhibited restrictions comparable to that of R1, thus not revealing a single domain conferring strong antiviral activity (Supplementary Figure S11B). Western blot analysis showed poor incorporation of the inactive cTM1 into virions (Supplementary Figure S11C). By comparison, the Env incorporation was largely unaffected by the chimeric constructs, in agreement with the published work [27]. We note that poor incorporation of the inactive cTM1 makes the comparative analysis of incorporation for this set of chimeric constructs less reliable.

To evaluate the ability of cTM1-derived chimeras to inhibit HIV-1 Env-mediated membrane fusion, HEK293T/17 effector cells transiently expressing HXB2 Env were co-cultured with TZM-bl target cells expressing CD4 and coreceptors. The cTM1/R1 chimeric constructs were transiently expressed in either effector or target cells. The inactive cTM1 showed no inhibition of cell-cell fusion, further supporting the correlation between negative imprinting and cell-cell fusion inhibitory activity. All chimeras, except cTM1-(N+C)R1, inhibited cell-cell fusion to an extent comparable to that of R1 (~2.5-fold, Supplementary Figure S11D). Collectively, our data suggest that IFITM-mediated negative imprinting is governed by interdomain cooperation rather than specific domains of these proteins, and that IFITM abundance or Env cleavage/incorporation are not the main determinants of this mode of HIV-1 restriction. Building on these insights, we next investigated whether intermolecular interactions could modulate virion-associated antiviral activity.

### 3.5. Hetero-Oligomers of Antiviral and Non-Antiviral IFITMs Retain the Ability to Negatively Imprint Virions

To determine whether negative imprinting of HIV-1 by hTM3 can be modulated by co-incorporation of a non-antiviral dTM1, these proteins were co-expressed in virus-producing HEK293T/17 cells, and the effect on viral infectivity was assessed. Cells were transfected with a fixed amount (0.5 μg) of HA-tagged dTM1 alone or in combination with varied amounts (0.1 or 0.5 μg) of HA-tagged hTM3.

**Figure 6:**
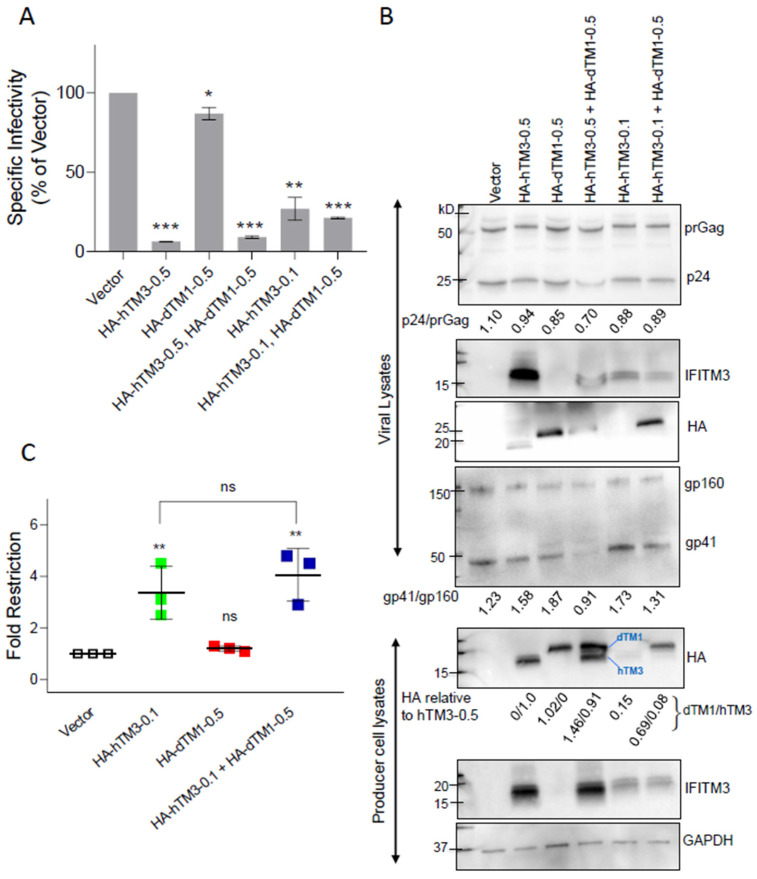
Human IFITM3’s negative imprinting activity is not modulated by co-incorporation of inactive dTM1. (**A**) A panel of HIV-1 pseudoviruses was produced in HEK293T/17 cells by co-transfection with plasmids encoding active HA-hTM3 and/or inactive HA-dTM1. Varying amounts of HA-hTM3 plasmid (0.1 or 0.5 μg) were used in combination with a fixed amount (0.5 μg) of HA-dTM1. Pseudovirus infectivity was measured in TZM-bl target cells at 48 h post-infection using a reporter luciferase assay. Specific infectivity was determined using p24-normalized virus input. Bar graphs represent means ± SD from two independent viral preparations. (**B**) Western blot analyses of the viral lysates showing virus production and Gag processing (p24 vs. prGag bands). Blots show HA-IFITM1, IFITM3, and Env incorporation into virions (top), along with IFITM expression levels in HEK293T/17 producer cell lysates (bottom). Densitometric analysis of HA blots is shown, with the band intensities normalized to HA-hTM3. The ratios of HA-hTM3 and HA-dTM1 band intensities are shown. Original Western Blot images are provided in Supplementary Figure S18. (**C**) A plot comparing fold-restriction of viral infectivity measured in the presence of HA-hTM3 or HA-dTM1 alone, and upon their co-expression for three independent viral preparations transfected with 0.1 μg HA-hTM3 and 0.5 μg HA-dTM1. Statistical significance was determined using Student’s *t*-test. ns (*p* > 0.05); * (*p* < 0.05); ** (*p* < 0.01); *** (*p* < 0.001).

Similar to FLAG-tagged hTM3 (Figure 1C), HA-hTM3 exhibited robust negative imprinting activity, reducing viral infectivity by 7.4 ± 1.0-fold (mean ± SD) at 0.5 μg and 3.9 ± 0.5-fold at 0.1 μg of transfected plasmid, respectively. By contrast, HA-dTM1 very modestly restricted the virus, as expected (Figure 6A). Notably, the infectivity of pseudoviruses containing both HA-hTM3 and HA-dTM1 was strongly reduced to the level observed with HA-hTM3 alone, even when only 0.1 μg of HA-hTM3 plasmid was co-expressed with 0.5 μg HA-dTM1. This finding implies that the non-antiviral ortholog does not interfere with the negative imprinting activity of hTM3. Efficient negative imprinting by HA-hTM3 in the presence of excess dTM1 was consistently observed across three independent viral preparations (Figure 6B,C). We note that Gag processing and Env incorporation were somewhat reduced for pseudoviruses produced by cells co-transfected with 0.5 μg of each HA-hTM3 and HA-dTM1 plasmids (Figure 6B and Figure S12A). To further assess the impact on Env incorporation at the single-particle level, we performed immunofluorescence staining of GFP-Vpr–labeled pseudovirions. Across the virus panel, single virions contained comparable levels of GFP-Vpr (Supplementary Figure S12B); however, we observed a reduced Env signal in virions produced by cells transfected with 0.5 μg of HA-hTM3 plasmid, consistent with our previous study [30], as well as in particles produced with 0.5 µg of each hTM3 and dTM1 plasmids (Supplementary Figure S12C). For this reason, we relied on the combination of 0.1 μg of hTM3 with 0.5 μg dTM1, which did not significantly perturb virus maturation or Env incorporation, for further analysis (Figure 6B and Figure S12A). Both HA-hTM3 and HA-dTM1 were robustly co-expressed in virus-producing cells, and dTM1 did not interfere with hTM3 expression in producer cells. However, incorporation of HA-hTM3 into virions was markedly diminished upon co-expression of HA-dTM1 (Figure 6B). Our results further highlight the lack of direct correlation between IFITM3 incorporation and antiviral activity.

**Figure 7:**
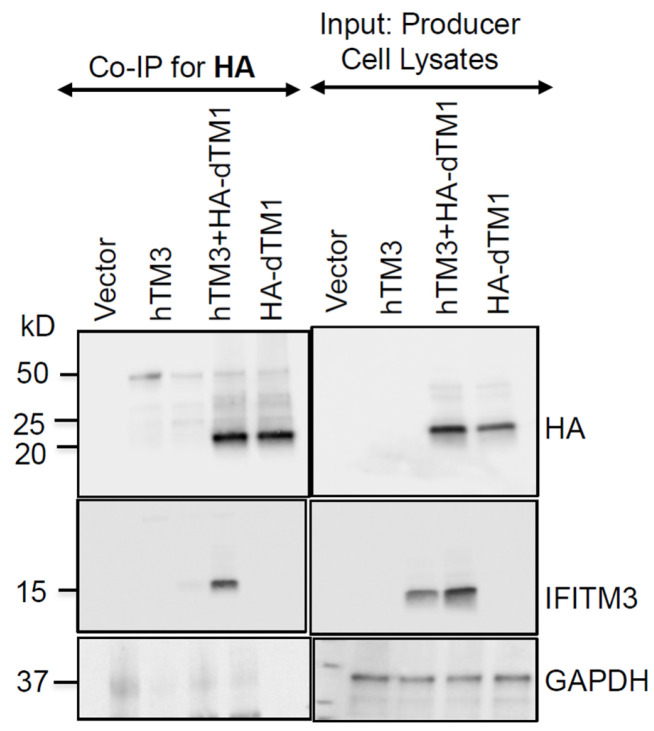
Co-expression and co-immunoprecipitation of antiviral and non-antiviral IFITMs. HEK293T/17 cells were co-transfected with equal amounts of plasmids encoding for antiviral hTM3 (untagged) and/or inactive HA-dTM1. Whole-cell lysates were collected, normalized for total protein content, and analyzed by Western blotting. Immunoblots show HA-dTM1 and hTM3 expression (“Input”, right). GAPDH served as a loading control. For co-immunoprecipitation, HA-dTM1 was pulled down using anti-HA antibodies, followed by SDS-PAGE and immunoblotting for IFITM3 (left). Original Western Blot images are provided in Supplementary Figure S19.

We next asked if the failure of dTM1 to modulate the negative imprinting activity of hTM3 is due to the inability of these proteins to interact/oligomerize. Toward this goal, we performed co-immunoprecipitation experiments with anti-HA antibodies using producer cell lysates containing HA-dTM1 and untagged hTM3, followed by SDS-PAGE and blotting for IFITM3 (Figure 7). Immunoblots showed the formation of hetero-oligomeric complexes between hTM3 and dTM1 that retain antiviral activity (Figure 6A,C). This finding, along with robust negative imprinting activity of G91L and G95L mutants (Figure 1C), suggests that negative imprinting by hTM3 may be independent of its homo-oligomerization, which has been implicated in its antiviral activity [8,9].

## 4. Discussion

Here, we assessed the determinants for the two modes of IFITMs’ anti-HIV-1 activity—target cell protection and negative imprinting of virions. Previous work reported a correlation between these two activities [20,27,44], although with a few notable exceptions involving mammalian orthologs [27]. It has also been documented that, whereas virus-incorporated IFITM3 potently reduced HIV-1 infectivity [20], target cell protection was more pronounced for low virus input, suggesting that this mode of restriction may be saturable. Unexpectedly, we found that several well-characterized hTM3 mutants, which failed to protect target cells against HIV-1 infection, incorporated into and robustly inhibited the infectivity of HIV-1 pseudoviruses. This observation, and the fact that these mutants also inhibit HIV-1 Env-mediated cell-cell and virus-cell fusion (Figure 3B,C), suggest that the mutant hTM3 proteins remain competent for both target cell protection and negative imprinting activities.

How can the inability to protect cells from infection be reconciled with the proposed anti-HIV-1 activity of the hTM3 mutants? Our immunostaining data indicate that these mutants’ failure to protect target cells is due to their mislocalization. Indeed, nearly all tested mutants exhibited an increased colocalization with the Golgi marker, similar to the previously reported block of the Golgi egress for the IFITM3 CIL mutants [42]. The Golgi retention, combined with the reduced expression levels of the mutants, can reduce their abundance at the sites of HIV-1 fusion. We note, however, that no significant changes in the G91L mutant colocalization with early or late endosomal markers were detected in [9], so further investigation of this mutant’s trafficking and localization is warranted. By comparison, the G95L mutant is well-expressed and distributed within the cell, like hTM3. Accordingly, this mutant exhibited a significant activity in all four functional assays—target cell protection, negative imprinting, virus-cell fusion, and cell-cell fusion. Collectively, our findings support the notion that the altered subcellular localization of otherwise active hTM3 mutants is responsible for the lack of target cell protection against HIV-1.

The discovery that several conserved IFITM3 motifs thought to be important for the antiviral phenotype are not essential for negative imprinting or inhibition of cell-cell fusion calls for reevaluation of the determinants of antiviral activity. These include the short conserved amphipathic helix (residues 59–68) [6,7,38] and residues involved in IFITM3 oligomerization (G91 and G95) [9]. We and others have shown that the amphipathic helix stiffens the cell membranes and reduces their propensity to undergo fusion [7,38]. However, robust negative imprinting of HIV-1 by the Δ59-68 mutant and the conservation of this helix among antiviral and non-antiviral mammalian IFITM orthologs (Supplementary Figure S13) argue against its role in negative imprinting by hTM3. Since the F67 residue within this helix has been implicated in cholesterol binding and target cell protection [38], our results indicate that cholesterol binding and membrane modification by the amphipathic helix may be dispensable for negative imprinting.

Our negative imprinting results suggest that hTM3 homo-oligomerization may also be dispensable for this mode of antiviral activity. Firstly, the lack of effect of the G91L and G95L mutations on negative imprinting of HIV-1 does not support the role of IFITM3 oligomerization in this mode of HIV-1 restriction. Secondly, we find that oligomers of active (hTM3) and inactive (dTM1) orthologs incorporated into virions retain antiviral activity (Figure 6). Note, however, that while G95L-mediated HIV-1 imprinting is in general agreement with the previous work, these authors have reported an attenuated G91L activity using this assay. We surmise that the difference in negative imprinting by G91L may be related to the levels of incorporation into virions.

In contrast to the robust negative imprinting of HIV-1 by all tested hTM3 mutants, the lack of this activity in dTM1 and cTM1 orthologs provided the opportunity to identify motifs critical for the antiviral activity through swapping distinct domains with antiviral orthologs. However, analyses of dTM1/D1Lb and cTM1/R1 chimeras did not reveal a single domain (or combinations of two domains) conferring the levels of negative imprinting to dTM1 or cTM1 variants that were close to those observed for D1Lb and R1, respectively. We note, however, that the conserved CIL domains of IFITM orthologs were not swapped, leaving the question regarding their functional roles open. Together, these findings further support the previous conclusion [27] that optimal negative imprinting is a result of cooperation between multiple IFITM domains.

Although negative imprinting did not strictly correlate with the IFITM incorporation or Env incorporation/cleavage, modest variations in these characteristics across chimeras can confound the interpretation of our functional results. A correlation between IFITM levels and both cell protection and negative imprinting by a panel of mammalian IFITM orthologs has been reported [20,27]. However, other studies, including our current work, did not observe such a correlation across different IFITM variants [26]. Of note, we detected a dose-dependent HIV-1 restriction by varying the expression/incorporation levels of hTM3 or the FLAG-Δ59–68 mutant (Supplementary Figure S5A,C). It thus appears that, while negative imprinting may be proportional to the level of incorporation for a given IFITM construct, no such correlation is generally observed across mutants and orthologs. In fact, one may argue that IFITM variants that strongly reduce HIV-1 infectivity, despite poor incorporation into virions, are gain-of-function mutations. These findings suggest that the level of IFITM incorporation into virions is not the only determinant for efficient negative imprinting.

The mechanism by which intraviral IFITMs reduce infectivity cannot be ascribed solely to the adverse effects on the HIV-1 Env cleavage or incorporation into virions, although altered levels of Env incorporation and cleavage in virions produced by IFITM3-expressing cells have been reported [21,23,45]. IFITMs also do not appear to regulate the spatial distribution of Env in virions, since we did not detect a significant disruption of the HIV-1 Env cluster formation in virions by IFITMs using super-resolution microscopy [30]. Our results may be interpreted as evidence that negative imprinting and target-cell protection activities may be mechanistically distinct. Alternatively, the striking phenotypic difference between the effects of IFITM mutations on cell protection and negative imprinting may arise from the exquisite sensitivity of the former activity to disruption of IFITM trafficking. Further studies are needed to uncover the mechanism of negative imprinting.

## 5. Conclusions

Our findings demonstrate that several human IFITM3 mutants, previously considered deficient in antiviral activity based on their failure to protect cells from HIV-1 infection, retain the ability to impair infectivity through incorporation into virions. This includes the human IFITM3 mutants lacking the conserved amphipathic helix and the oligomerization motif that are critical for cell protection against HIV-1 infection. Thus, the lack of antiviral activity by these mutants in a target cell protection assay is not due to a loss of antiviral function but rather a result of altered intracellular localization. Our results also suggest that negative imprinting of HIV-1 depends on the coordinated contribution of multiple IFITM domains rather than a single domain. Together, these findings support the notion that negative imprinting and target cell protection can be mechanistically distinct, with the latter being strongly modulated by IFITM trafficking and subcellular localization.

## Data Availability

The original contributions presented in this study are included in the article and its Supplementary Material. Further inquiries can be directed to the corresponding author.

## Supplementary Materials

The following supporting information can be downloaded at: https://www.mdpi.com/article/10.3390/biom16030459/s1: Figure S1: Negative imprinting by FLAG-tagged and untagged hTM3 mutants; Figure S2: Target cell protection against pseudoviruses carrying non-HIV-1 fusion glycoprotein; Figure S3: Negative imprinting of hTM3-resistant HIV-1 AD8 Env; Figure S4: Immunoblotting analysis of the independent HXB2 HIV-1 pseudovirus panel; Figure S5: Dose-dependence of HIV-1 negative imprinting by the hTM3 and FLAG-Δ59-68 mutant; Figure S6: hTM3 expression does not alter CD4 or CXCR4 coreceptor expression; Figure S7: Analysis of IFITM3–GM130 colocalization in HEK293 cells expressing CD4, CCR5 and hTM3 and its mutants using Pearson’s overlap coefficient; Figure S8: The effect of FLAG-tagging and CsA treatment on abundance and subcellular localization of hTM3 and its mutants; Figure S9: hTM3 mutants display reduced abundance and altered subcellular localization in TX-100-permeabilized cells; Figure S10: Virus-incorporated dog and cat IFITM1 orthologs fail to restrict HIV-1 infectivity; Figure S11: Negative imprinting of HIV-1 by chimeras between antiviral and non-antiviral IFITM1 orthologs; Figure S12: Analysis of HIV-1 Env incorporation into virions; Figure S13: Amphipathicity of the IFITM helix is conserved across antiviral and non-antiviral orthologs; Figures S14–S19: Original, uncropped Western Blot images for the target cell lysates.
