# Supplementary material for: Divergent HIV-1 Restriction Phenotypes of IFITMs Expressed in Target Cells and Incorporated into Virions
Source: Biomolecules. 2026 Mar 18;16(3):459. doi: 10.3390/biom16030459 (PMC13023901; doi:10.3390/biom16030459)
Supplement: Supplementary file 1 [file biomolecules-16-00459-s001.zip › biomolecules-4178069-supplementary.pdf]

# Divergent HIV-1 restriction phenotypes of IFITMs expressed in target cells and incorporated into virions

Smita Verma, David Prikryl, Mariana Marin, Ruben Markosyan, Andrea Cimarelli, and Gregory B. Melikyan

## Supplementary Figures and Legends

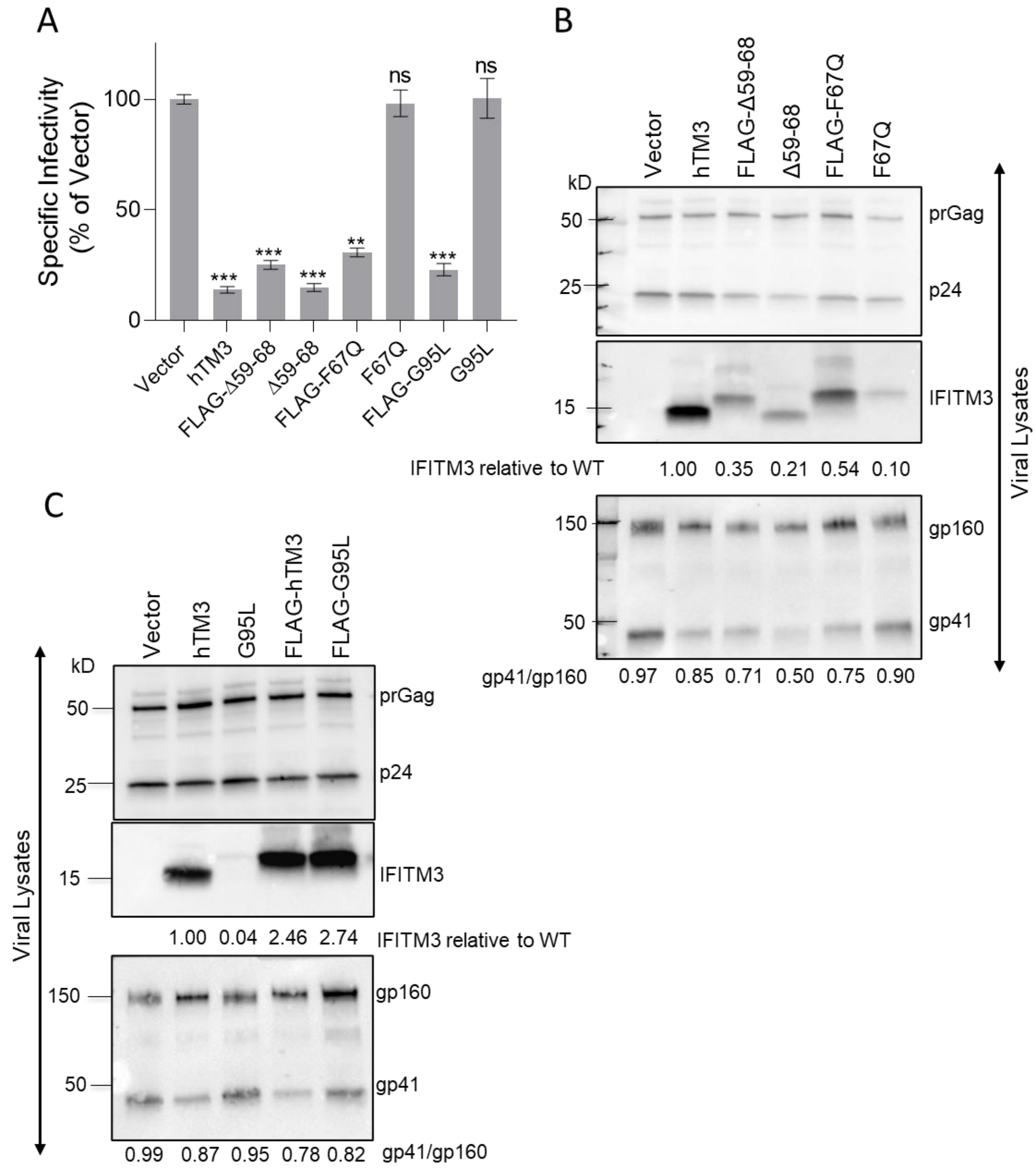


**Suppl. Fig. S1: Negative imprinting by FLAG-tagged and untagged hTM3 mutants:** (A) Specific infectivity of HXB2 Env–bearing pseudoviruses containing FLAG-tagged and untagged hTM3 and its mutant proteins measured in TZM-bl cells infected with equal amounts of virus based upon the p24 content. The resulting luciferase signal is plotted as % of vector. Data represent means ± SD from two independent experiments. (B, C) Western blots showing Gag processing, IFITM3 incorporation and Env incorporation/cleavage in viral lysates, respectively, for the FLAG-tagged and untagged hTM3 mutants.

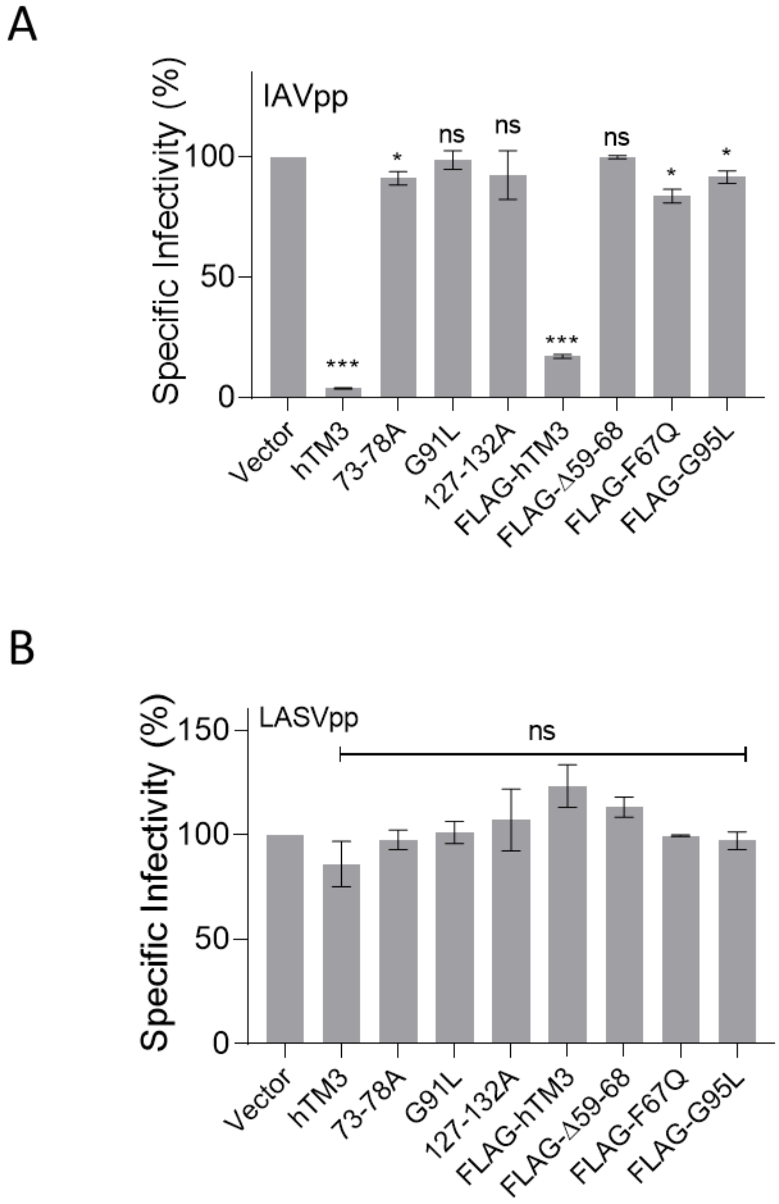


**Suppl. Fig. S2: Target cell protection against pseudoviruses carrying non-HIV-1 fusion glycoprotein.** Assay for infection of HEK293.CD4.CCR5 cells expressing hTM3 mutants using luciferase reporter NL4-3 HIV-1 particles pseudotyped with the IAV HA (A) or the Lassa virus glycoprotein (LASV) (B). Infectivity was quantified at 48 h post-infection and normalized to the vector control. Data represent means ± SD of triplicate experiments.

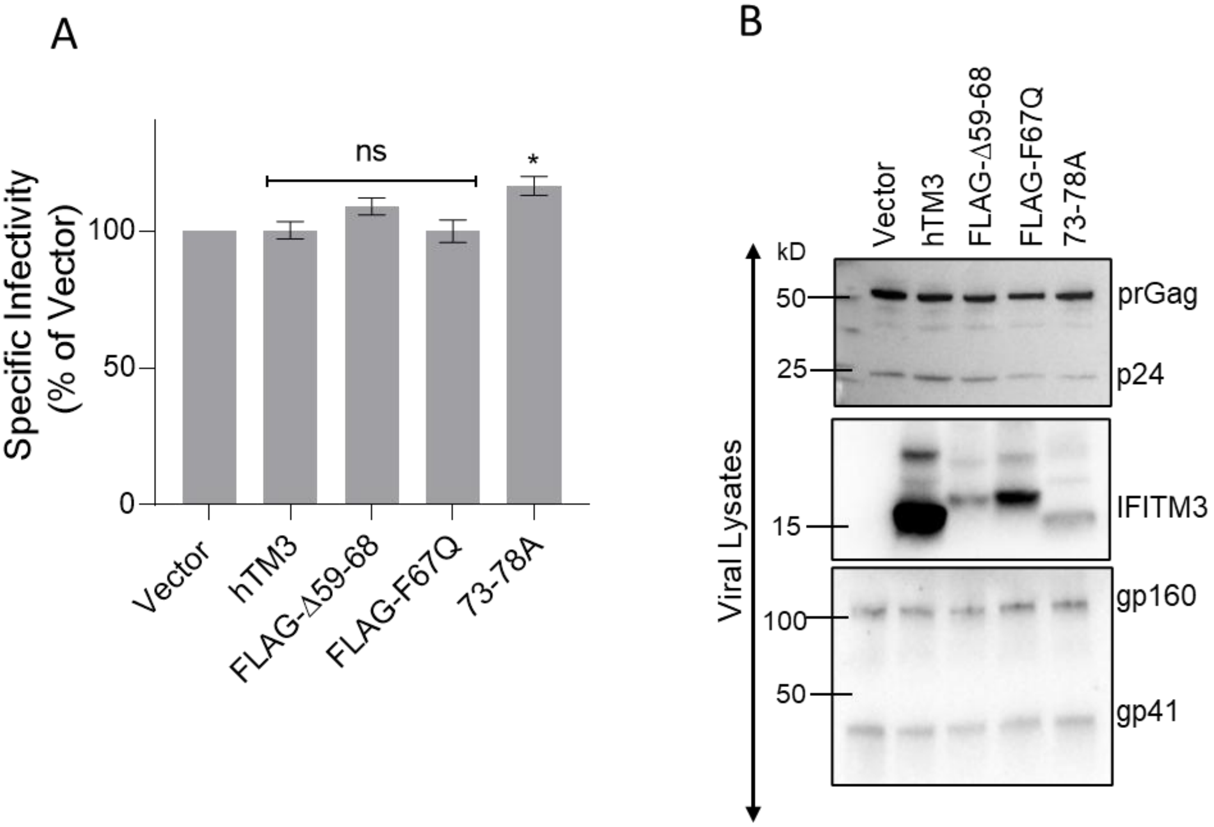


**Suppl. Fig. S3: Negative imprinting of hTM3-resistant HIV-1 AD8 Env.** (A) Specific infectivity of IFITM3-resistant AD8 Env–bearing pseudoviruses containing hTM3 and selected mutant proteins measured in TZM-bl cells. The resulting luciferase signal was normalized to the viral p24 content and plotted as % of vector. Data represent means ± SD from two independent experiments. (B) Western blots showing Gag processing, IFITM3 incorporation and Env incorporation/cleavage in viral lysates.

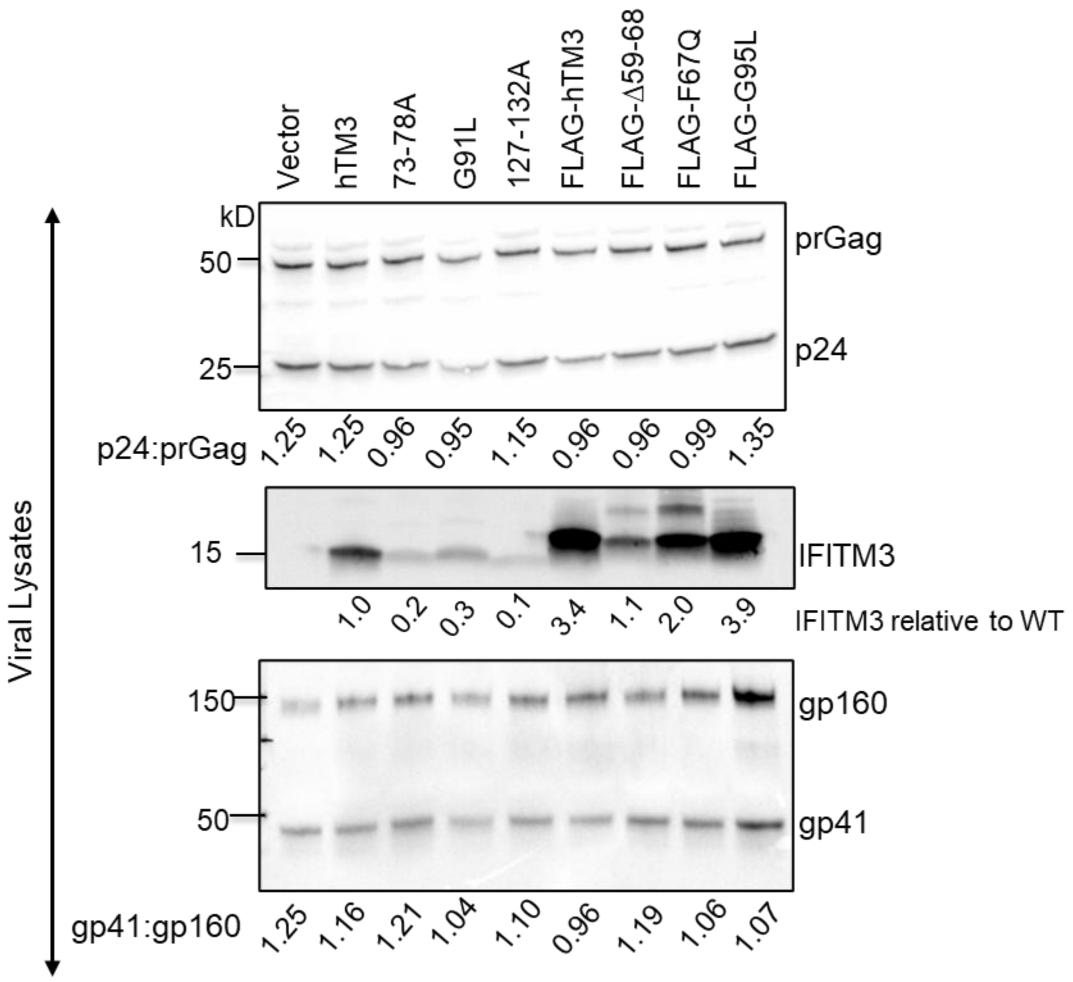


**Suppl. Fig. S4: Immunoblotting analysis of the independent HXB2 HIV-1 pseudovirus panel (see Fig. 1).** Shown are Gag processing, IFITM3 incorporation and Env incorporation/cleavage in viral lysates.

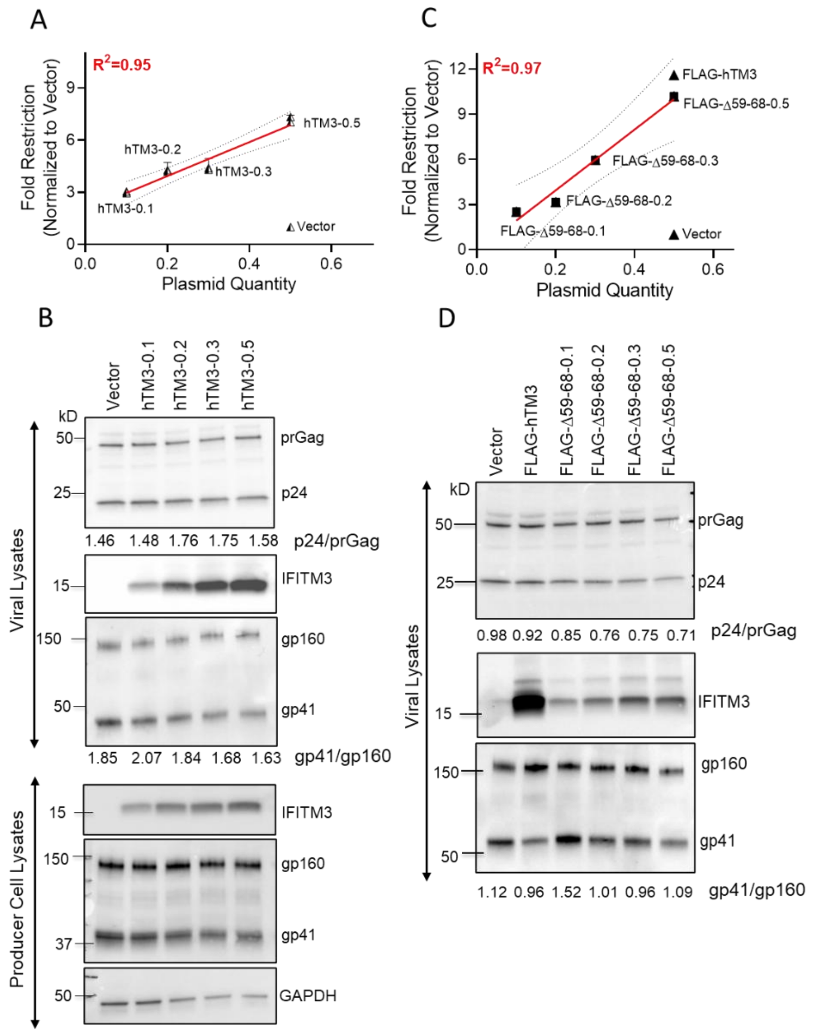


**Suppl. Fig. S5: Dose-dependence of HIV-1 negative imprinting by the hTM3 and FLAG-Δ59-68 mutant.** (A, C) HIV-1 HXB2 Env pseudoviruses were produced by co-transfecting increasing amounts of plasmid encoding for the hTM3 (A) and FLAG-Δ59-68 mutant (0.1-0.5 µg) and 0.5 µg of FLAG-hTM3 plasmid (C), as a control respectively. The infectivity of produced pseudoviruses was measured using a luciferase reporter assay and normalized to the viral p24 content. Data are means ± SD from two independent experiments. Fold restriction values (normalized to vector control) were plotted against plasmid quantity for hTM3 (A) and FLAG-Δ59-68 mutant (B). Linear regressions (red lines) and R² are shown for all points excluding the vector control. Dashed lines indicate the 95% confidence interval. (B, D) Immunoblots of virions produced with increasing amounts of hTM3 (B) and the FLAG-Δ59-68 mutant (D) plasmid showing the HIV-1 Gag processing, IFITM incorporation and Env cleavage (gp41/gp160) into virions.

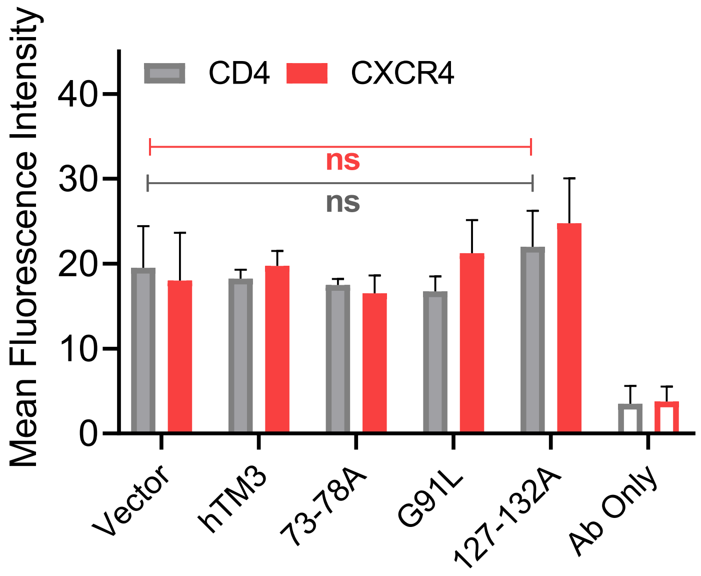


**Suppl. Fig. S6: hTM3 expression does not alter CD4 or CXCR4 coreceptor expression.** Target cells expressing CD4 or CXCR4 and hTM3 constructs or control cells were harvested and incubated with primary antibodies against CD4 or CXCR4 for 45 min on ice. Cells were washed, fixed, incubated with respective fluorescently labeled secondary antibodies, and analyzed by flow cytometry. The expression levels of proteins were plotted as mean ± SD of mean fluorescence intensity (MFI) from two independent experiments. Statistical significance was calculated using Student's t-test for each hTM3 mutant relative to the respective vector control for both CD4 and CXCR4 expression, shown in gray and red, respectively.

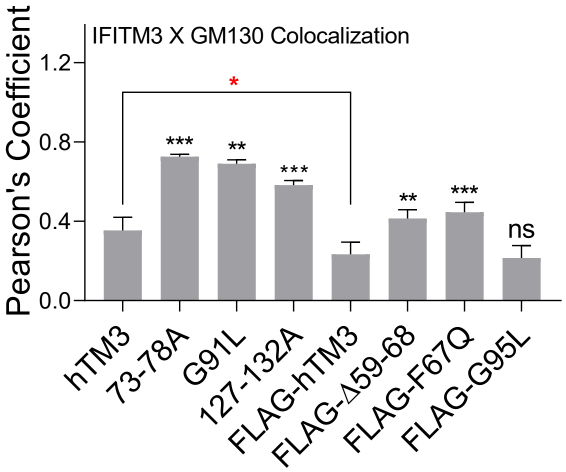


**Suppl. Fig. S7: Analysis of IFITM3–GM130 colocalization in HEK293 cells expressing CD4, CCR5 and hTM3 and its mutants using Pearson's overlap coefficient.** Statistical analyses were performed by comparing untagged mutants to untagged hTM3 and FLAG-tagged mutants to FLAG-hTM3 (black asterisks) using Student's t-test. The significance level for untagged vs FLAG-tagged hTM3 (red asterisks) is also shown.

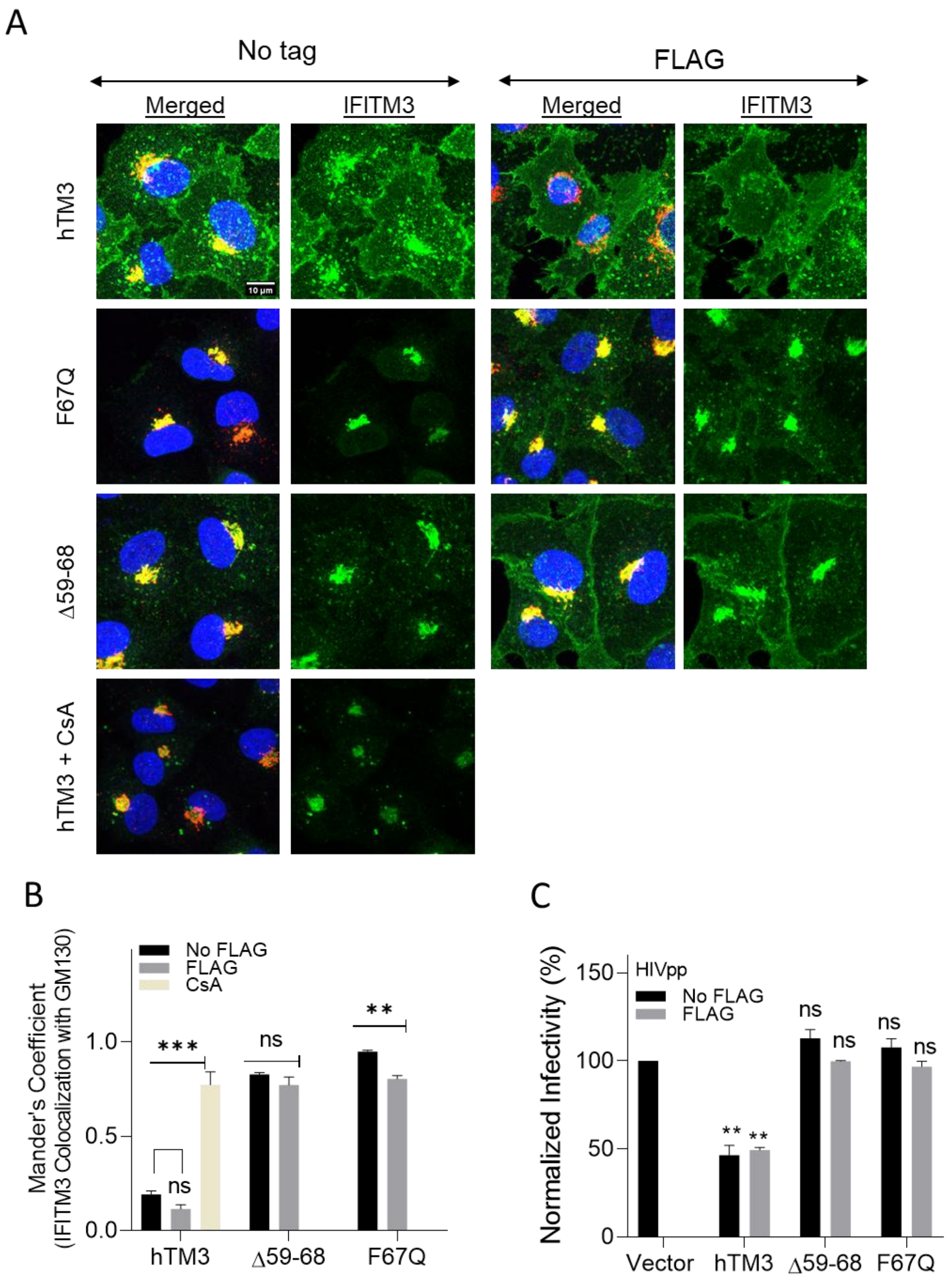


**Suppl. Fig. S8: The effect of FLAG-tagging and CsA treatment on abundance and subcellular localization of hTM3 and its mutants.** (A) A549 cells stably expressing the indicated hTM3 or its mutants (FLAG-tagged or untagged) were treated with DMSO or CsA (20 μM) for 90 min, fixed, permeabilized with digitonin, and immunostained with anti-IFITM3 (green) and anti-

GM130 (Golgi marker, red) antibodies. Nuclei were counterstained with Hoechst 33342 (blue). Scale bar, 10 µm. (B) Quantitative analysis of IFITM3 expression levels and colocalization with GM130 was performed using Mander's overlap coefficient. FLAG-tagged and untagged mutants were compared, including conditions with CsA treatment as indicated. Statistical significance was determined using Student's t-test. (C) Target cell protection against HIV-1 pseudovirus infection was tested using HEK293.CD4.CCR5 cells expressing FLAG-tagged and untagged mutants. Infectivity was quantified 48 h post-infection and normalized to the vector control. Data represent means ± SD of duplicate experiments. Statistical significance: n.s. ($p > 0.05$); * ($p < 0.05$); *** ($p < 0.001$).

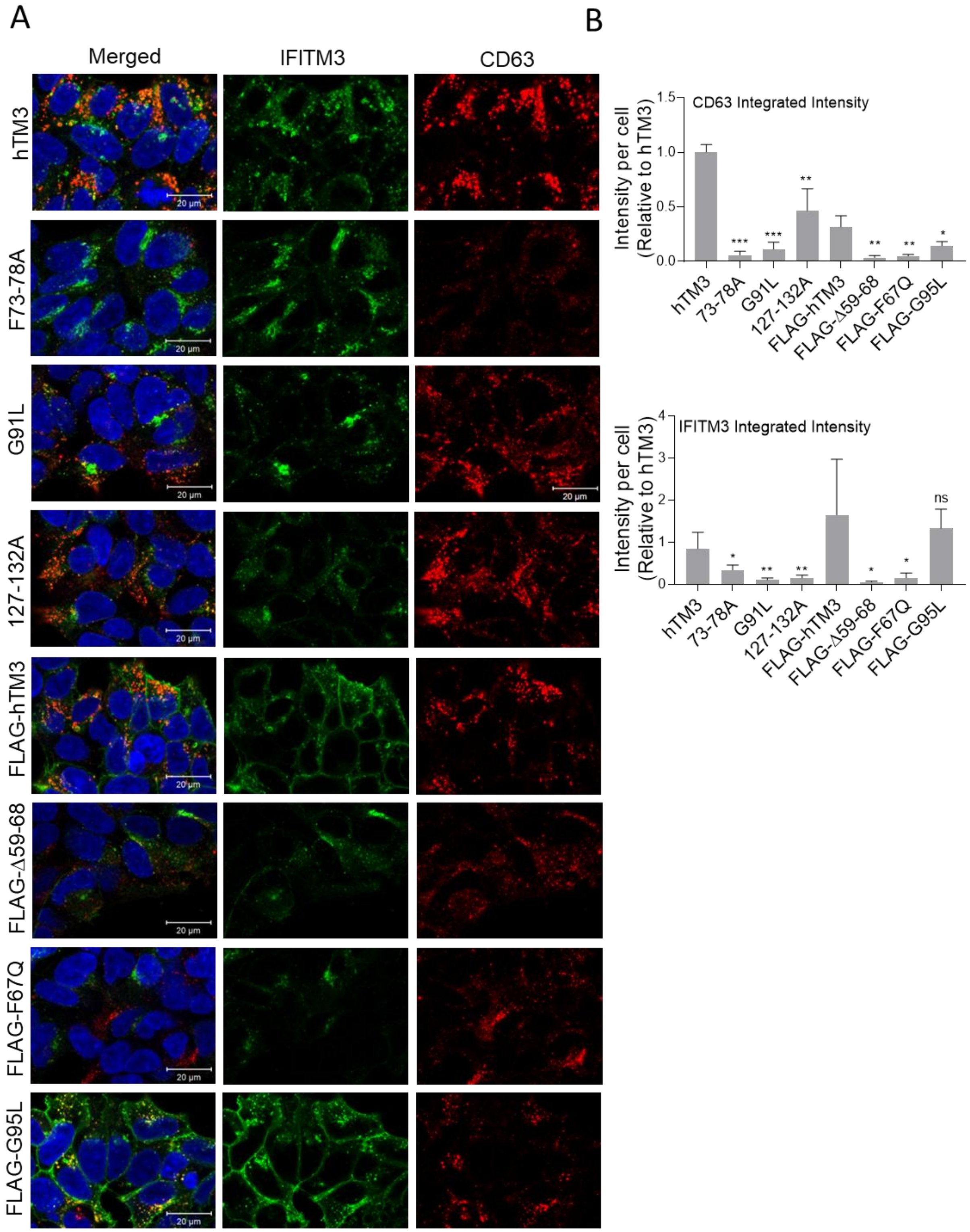


**Suppl. Fig. S9: hTM3 mutants display reduced abundance and altered subcellular localization in TX-100-permeabilized cells.** (A) HEK293.CD4.CCR5 cells stably

expressing hTM3 or the indicated hTM3 mutants were fixed, permeabilized with triton (TX-100), and immunostained with anti-IFITM3 (green) and anti-CD63 (late endosomes, red) antibodies. Scale bar: 20 µm. (B) Integrated fluorescence intensity per cell was measured for IFITM3 or CD63 and normalized to hTM3 levels. Statistical analyses were performed by comparing untagged mutants to untagged hTM3 and FLAG-tagged mutants to FLAG-hTM3 (black asterisks). Data represents mean ± SD from multiple fields of view across two independent experiments.

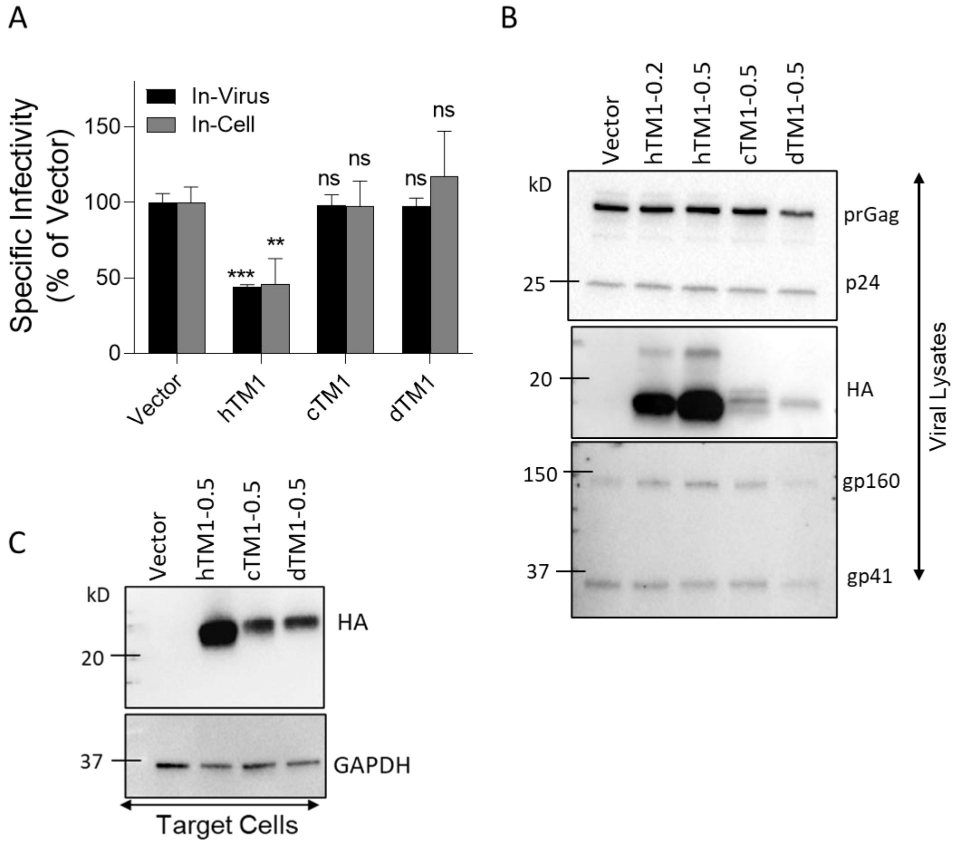


**Suppl. Fig. S10: Virus-incorporated dog and cat IFITM1 orthologs fail to restrict HIV-1 infectivity.** (A) Analysis of two HIV-1 restriction modes of IFITM1 orthologs – target cell protection ("In-Cell") and negative imprinting ("In-Virus"). HIV-1 pseudoviruses were produced in HEK293T/17 cells co-transfected with plasmids encoding for the indicated IFITM1 orthologs. For comparison, different amounts of HA-hIFITM1 (hTM1) plasmids (0.2 and 0.5 µg) were used for transfection of virus-producing cells. Pseudovirus infectivity (normalized to p24 content) was measured in TZM-bl cells at 48 h post-infection using a luciferase assay. Target cell protection against HIV-1 pseudovirus infection was tested using HEK293.CD4.CCR5 cells. Relative infectivity was normalized to the vector control. Bar graphs represent means ± SD from two independent viral preparations. (B) Western blot analyses of the Gag cleavage, Env processing and incorporation into virions, and IFITM1 incorporation (using anti-HA antibody) in viral lysates. (C) Expression levels of the IFITM1 orthologs in transduced HEK293.CD4.CCR5 cells. Cell lysates were analyzed by immunoblotting with anti-HA and anti-GAPDH (loading control) antibodies.

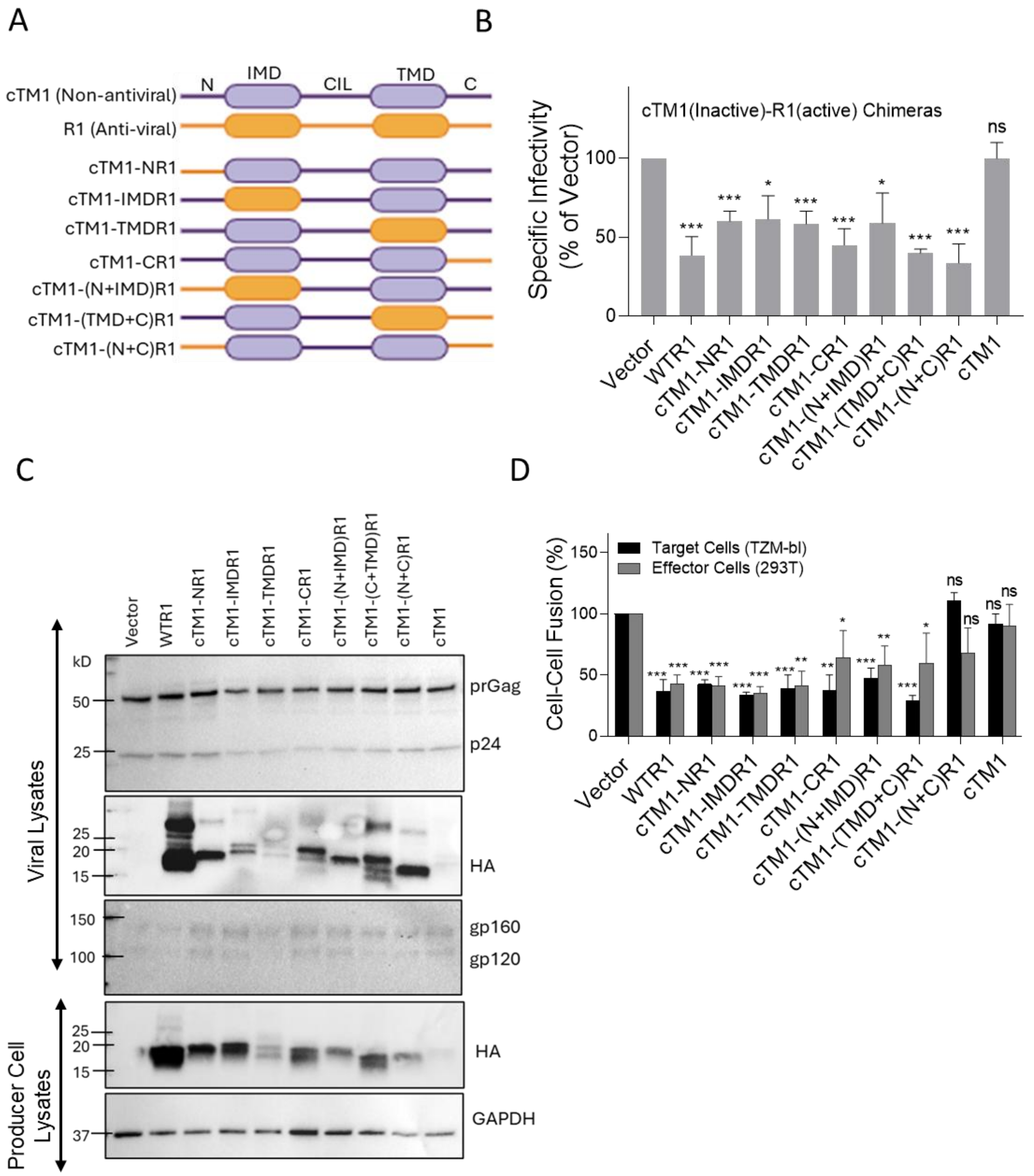


**Suppl. Fig. S11: Negative imprinting of HIV-1 by chimeras between antiviral and non-antiviral IFITM1 orthologs.** (A) Schematic representation of the chimeras with swapped domains of cat cTM1 (non-antiviral) and rabbit R1 (antiviral) IFITM1 proteins. (B) Normalized infectivity of HIV-1 pseudoviruses carrying the indicated IFITM1 cTM1/R1 chimeras. TZM-bl cells were infected for 48 h with pseudovirions produced by HEK293T/17 cells transfected with the respective chimeric constructs. Infectivity was measured using a luciferase assay and normalized to the vector control. Data represent means ± SD of three independent viral

preparations. Statistical significance: n.s. ($p > 0.05$); * ($p < 0.05$); *** ($p < 0.001$). (C) Western blotting analysis of viral lysates showing Gag processing, HIV-1 Env cleavage and incorporation into virions, and IFITM1 incorporation into virions (top). HA-IFITM1 expression levels of the chimeric constructs in the HEK293T/17 producer cell lysates are also shown (bottom). GAPDH was used as a loading control. (D) HEK293T/17 cells transiently expressing HIV-1 Env (HXB2 strain) were fused to TZM-bl cells expressing CD4 and coreceptors. Cat/rabbit IFITM1 chimeras were expressed by transfection of either effector HEK293T/17 cells (gray bars) or target TZM-bl cells (black bars). Effector and target cells were loaded with different cytoplasmic dyes, co-cultured for 2 h at 37 ºC, and the extent of fusion (fraction of double-positive cells) was measured by fluorescence microscopy.

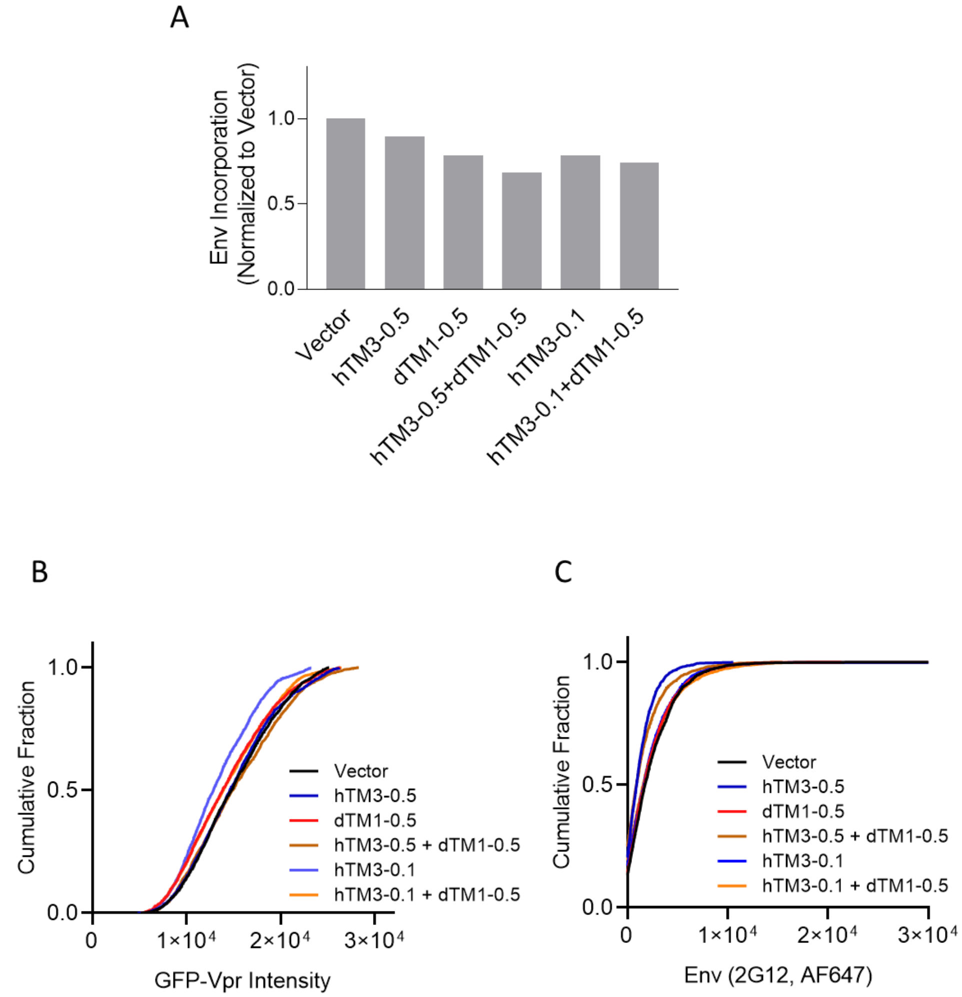


**Suppl. Fig. S12: Analysis of HIV-1 Env incorporation into virions.** (A) Analysis of Env incorporation into virions based on Western blotting data in Fig. 6B. Env incorporation was measured by densitometry through calculating the ratio of the total Env bands (gp41+gp160) over the sum of p24 and p55 bands. (B, C) HIV-1 particles pseudotyped with HXB2 Env were labeled with GFP-Vpr, as described in Methods. Single particle analysis of GFP-Vpr incorporation (B) and immunofluorescence signal reporting HIV-1 Env incorporation into virions using the anti-gp120 2G12 antibody and anti-human AF647-conjugated secondary antibody.

A

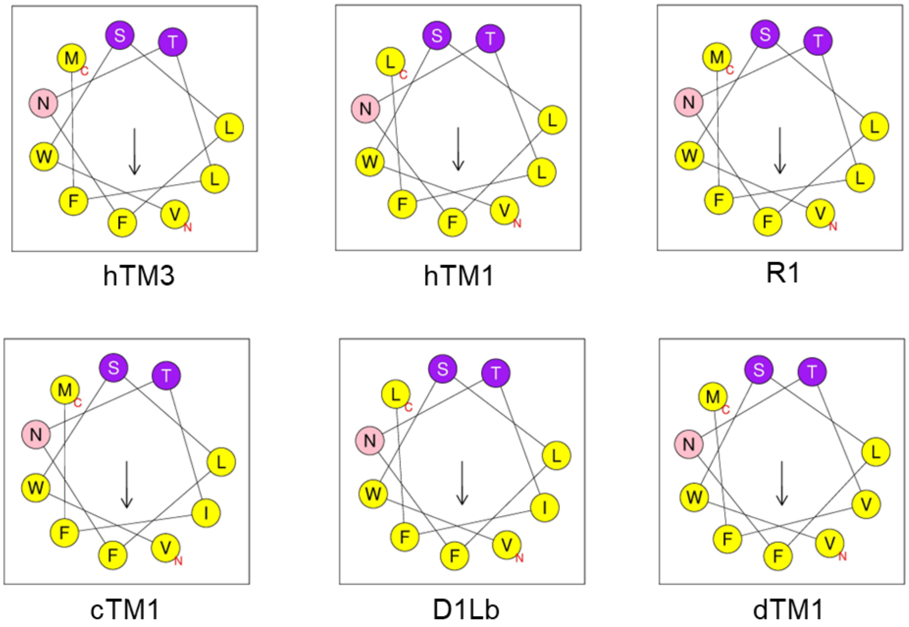


B

| Peptide | Mean Hydrophobic Moment | Hydrophobicity |
|---|---|---|
| hTM3 | 0.479 | 1.130 |
| hTM1 | 0.439 | 1.177 |
| R1 (active) | 0.483 | 1.130 |
| cTM1 (inactive) | 0.485 | 1.140 |
| D1Lb (active) | 0.445 | 1.187 |
| dTM1 (inactive) | 0.450 | 1.082 |

**Suppl. Fig. S13: Amphipathicity of the IFITM helix is conserved across antiviral and non-antiviral orthologs.** (A) Helical wheel projections of the key amphipathic helix IFITM residues (59–68) generated using HELIQUEST highlight the sequence conservation and similar helix amphipathicity across antiviral and non-antiviral IFITMs. Hydrophobic residues are shown in yellow, hydrophilic residues in pink or purple, and arrows depict the magnitude and orientation of the mean hydrophobic moment. (B) Mean hydrophobic moment and hydrophobicity values are expressed in arbitrary units with higher values indicating greater amphipathicity.

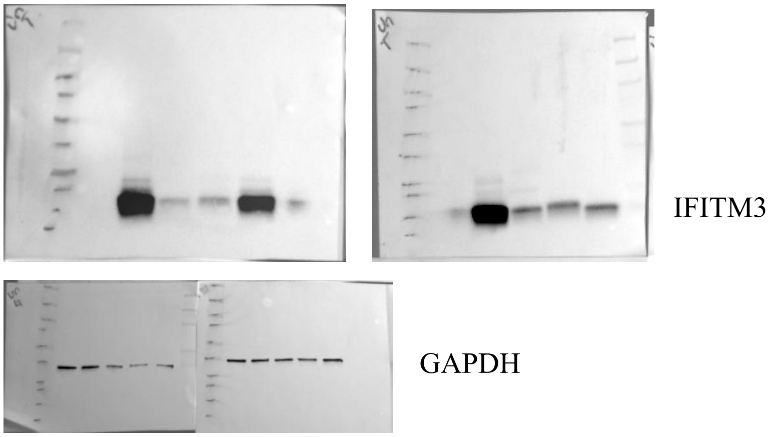


**Suppl. Fig. S14: Original, uncropped Western Blot images for the target cell lysates Fig. 1D**

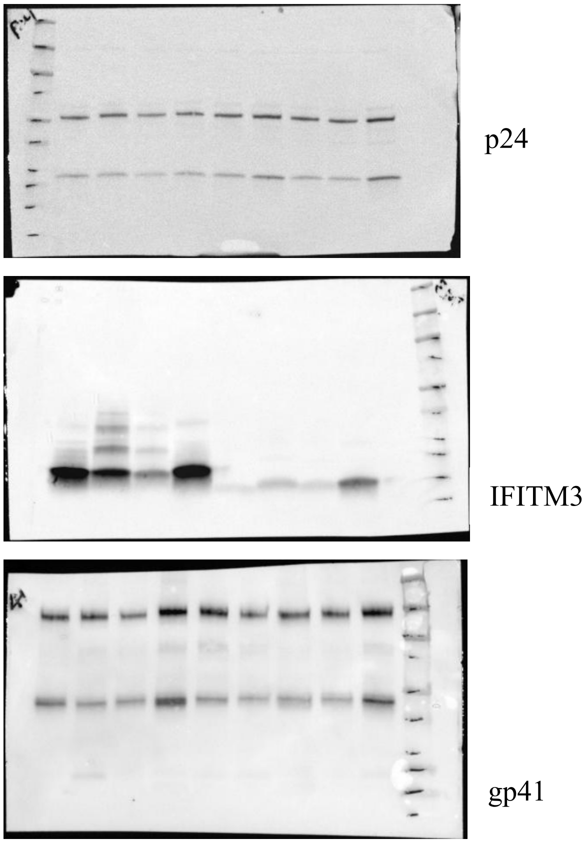


**Suppl. Fig. S15: Original, uncropped Western Blot images for the viral lysates Fig. 1E**

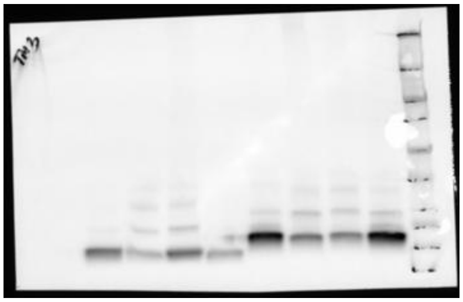

IFITM3

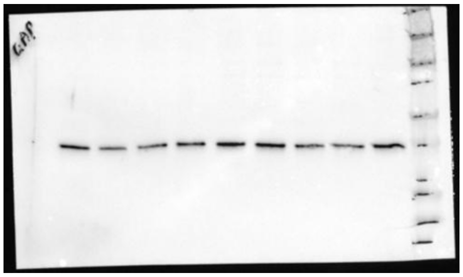

GAPDH

**Suppl. Fig. S16: Original, uncropped Western Blot images for the producer cell lysates Fig. 1F**

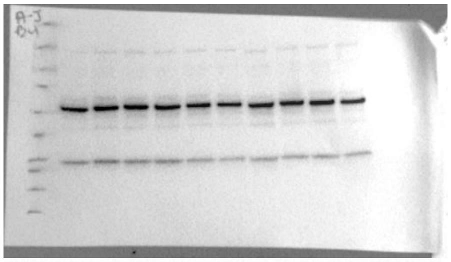

p24

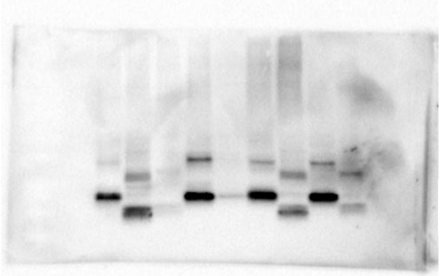

HA (in virions)

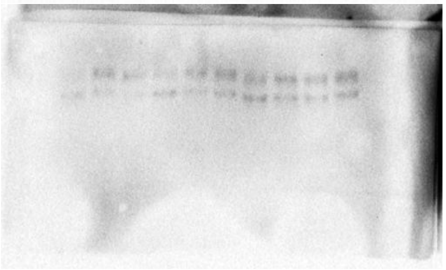

gp120

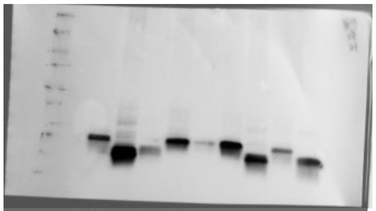

HA (in producer cell lysates)

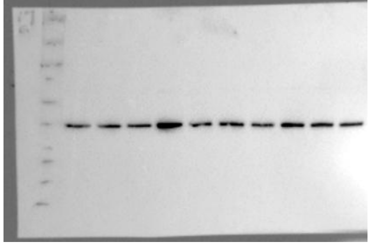

GAPDH

**Suppl. Fig. S17: Original, uncropped Western Blot images for the viral and producer cell lysates Fig. 5C**

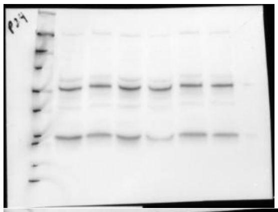

p24

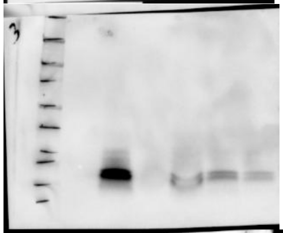

IFITM3 (in virions)

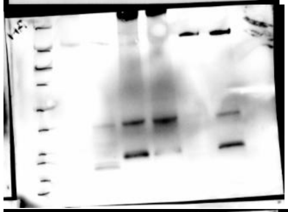

HA (in virions)

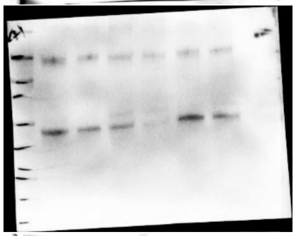

gp41

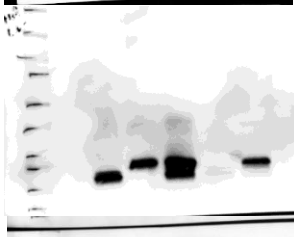

HA (in producer cell lysates)

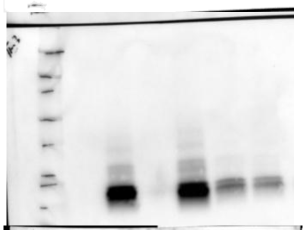

IFITM3 (in producer cell lysates)

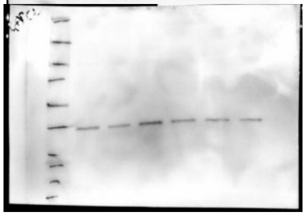

GAPDH

**Suppl. Fig. S18: Original, uncropped Western Blot images for the viral and producer cell lysates Fig. 6B**

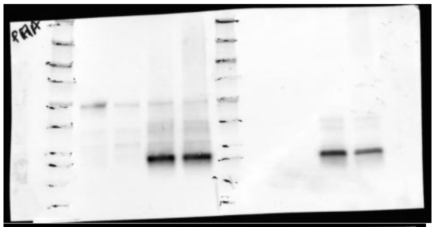

HA (blot's left-Co-IP; blot's right-Input producer cell lysates)

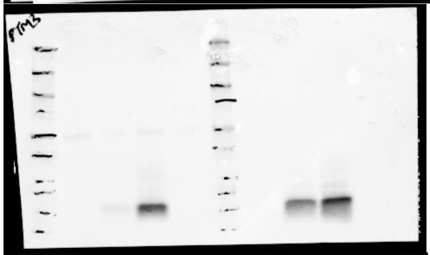

IFITM3 (blot's left-Co-IP; blot's right-Input producer cell lysates)

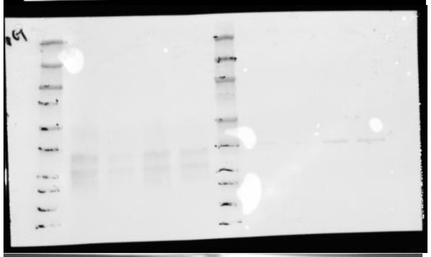

GAPDH (blot's left-Co-IP)

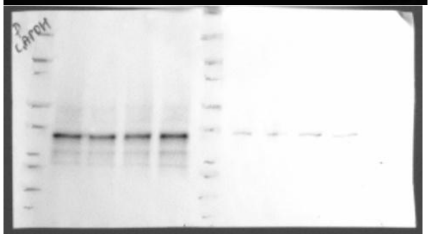

GAPDH (blot's left-Input producer cell lysate)

**Suppl. Fig. S19: Original, uncropped Western Blot images for the Fig. 7**
